# Cellular and humoral immune responses following SARS-CoV-2 mRNA vaccination in patients with multiple sclerosis on anti-CD20 therapy

**DOI:** 10.1038/s41591-021-01507-2

**Published:** 2021-09-14

**Authors:** Sokratis A. Apostolidis, Mihir Kakara, Mark M. Painter, Rishi R. Goel, Divij Mathew, Kerry Lenzi, Ayman Rezk, Kristina R. Patterson, Diego A. Espinoza, Jessy C. Kadri, Daniel M. Markowitz, Clyde E. Markowitz, Ina Mexhitaj, Dina Jacobs, Allison Babb, Michael R. Betts, Eline T. Luning Prak, Daniela Weiskopf, Alba Grifoni, Kendall A. Lundgreen, Sigrid Gouma, Alessandro Sette, Paul Bates, Scott E. Hensley, Allison R. Greenplate, E. John Wherry, Rui Li, Amit Bar-Or

**Affiliations:** 1 Institute for Immunology, University of Pennsylvania Perelman School of Medicine, Philadelphia, PA USA; 2 Division of Rheumatology, Department of Medicine, University of Pennsylvania Perelman School of Medicine, Philadelphia, PA USA; 3 Immune Health, University of Pennsylvania Perelman School of Medicine, Philadelphia, PA USA; 4 Center for Neuroinflammation and Experimental Therapeutics, University of Pennsylvania Perelman School of Medicine, Philadelphia, PA USA; 5 Department of Neurology, University of Pennsylvania Perelman School of Medicine, Philadelphia, PA USA; 6 Department of Systems Pharmacology and Translational Therapeutics, University of Pennsylvania Perelman School of Medicine, Philadelphia, PA USA; 7 Immunology Graduate Group, Perelman School of Medicine, University of Pennsylvania, Philadelphia, PA USA; 8 Department of Microbiology, University of Pennsylvania Perelman School of Medicine, Philadelphia, PA USA; 9 Department of Pathology and Laboratory Medicine, University of Pennsylvania Perelman School of Medicine, Philadelphia, PA USA; 10 Center for Infectious Disease and Vaccine Research, La Jolla Institute for Immunology, La Jolla, CA USA; 11 Penn Center for Research on Coronavirus and Other Emerging Pathogens, University of Pennsylvania Perelman School of Medicine, Philadelphia, PA USA; 12 Department of Medicine, Division of Infectious Diseases and Global Public Health, University of California San Diego, La Jolla, CA USA; 13 Parker Institute for Cancer Immunotherapy, University of Pennsylvania Perelman School of Medicine, Philadelphia, PA USA

**Keywords:** Multiple sclerosis, RNA vaccines, T cells, Antibodies, SARS-CoV-2

## Abstract

SARS-CoV-2 messenger RNA vaccination in healthy individuals generates immune protection against COVID-19. However, little is known about SARS-CoV-2 mRNA vaccine-induced responses in immunosuppressed patients. We investigated induction of antigen-specific antibody, B cell and T cell responses longitudinally in patients with multiple sclerosis (MS) on anti-CD20 antibody monotherapy (*n* = 20) compared with healthy controls (*n* = 10) after BNT162b2 or mRNA-1273 mRNA vaccination. Treatment with anti-CD20 monoclonal antibody (aCD20) significantly reduced spike-specific and receptor-binding domain (RBD)-specific antibody and memory B cell responses in most patients, an effect ameliorated with longer duration from last aCD20 treatment and extent of B cell reconstitution. By contrast, all patients with MS treated with aCD20 generated antigen-specific CD4 and CD8 T cell responses after vaccination. Treatment with aCD20 skewed responses, compromising circulating follicular helper T (T_FH_) cell responses and augmenting CD8 T cell induction, while preserving type 1 helper T (T_H_1) cell priming. Patients with MS treated with aCD20 lacking anti-RBD IgG had the most severe defect in circulating T_FH_ responses and more robust CD8 T cell responses. These data define the nature of the SARS-CoV-2 vaccine-induced immune landscape in aCD20-treated patients and provide insights into coordinated mRNA vaccine-induced immune responses in humans. Our findings have implications for clinical decision-making and public health policy for immunosuppressed patients including those treated with aCD20.

## Abstract (web summary)

SARS-CoV-2-specific antibodies and memory B cells are significantly reduced, but CD4^+^ and CD8^+^ T cells are robustly activated, in patients with multiple sclerosis on anti-CD20 monotherapy versus healthy controls after BNT162b2 or mRNA-1273 mRNA vaccination.

## Main

Coronavirus disease 19 (COVID-19) has caused a global pandemic with profound public health and socioeconomic sequelae due to the absence of protective immunity to SARS-CoV-2, the viral infectious cause of COVID-19 (refs. ^1,2^). Vaccines were rapidly developed with the goals of protecting individuals and achieving herd immunity^3^. The two mRNA vaccines granted Food and Drug Administration Emergency Use Authorization in the US, BNT162b2 (Pfizer-BioNTech) and mRNA-1273 (Moderna), were shown in phase 3 clinical trials of healthy individuals to be highly effective in preventing moderate-to-severe COVID-19 (refs. ^4,5^). Individuals with underlying autoimmune disorders, including MS, and those on immune-modulatory therapies were not included in these trials. As a result, the magnitude and quality of the immune response to mRNA vaccination is not well characterized in these potentially vulnerable patients who may be at greater risk for COVID-19-associated morbidity and mortality and more prone to infect others^6–12^.

aCD20-based B cell-depleting strategies are implemented in hematological malignancies^13^ and a variety of autoimmune disorders^14^, including MS^15,16^. On antigen exposure, B cells can form memory B cells or differentiate into plasmablasts and plasma cells^17^. As a result, vaccine-specific antibody responses are diminished in patients on aCD20 therapy^18–23^. For SARS-CoV-2 mRNA vaccination, B cell depletion results in decreased spike-specific antibodies in patients with chronic inflammatory diseases^24^, including patients with MS^25^. However, the kinetics of antibody responses and their relationship to peripheral B cell depletion and spike-specific memory B cells are poorly understood.

The role of B cells in T cell priming, differentiation and proliferation is unclear, especially in humans. Some studies suggest that B cells are not required for T cell responses^26–28^ whereas other work supports a role for B cells as antigen-presenting cells that facilitate T cell priming^29–35^. In COVID-19, CD4 and CD8 T cell immunity is generated with T cell responses correlating with better outcomes in some settings^36–38^. Robust CD8 T cell responses are associated with improved survival in COVID-19 patients with hematologic malignancies, including patients on therapies that deplete B cells^39^. These data suggest that T cells may provide protective immunity and limit severe disease in settings where antibody responses are lacking. In addition, T cells are capable of recognizing mutant SARS-CoV-2 variants^40,41^ that can partially escape humoral-based immunity. Despite these data, the induction of T cell responses by mRNA vaccination in patients on B cell-depleting therapies is poorly understood.

In this study, we analyzed patients with MS to evaluate the effect of aCD20 therapy on SARS-CoV-2 mRNA vaccine responses. Although most patients with MS treated with aCD20 (MS-aCD20) made detectable spike-binding antibodies and 50% made RBD antibodies, antibody titers were lower, delayed and had reduced neutralizing activity compared with healthy controls. All patients with MS treated with aCD20 developed spike-specific CD4 T cell responses and enhanced CD8 T cell responses. Finally, comparing patients with MS treated with aCD20 who did and did not generate anti-RBD IgG responses revealed differences in immune response coordination, with substantial reduction in vaccine-induced circulating T_FH_ cell responses and reciprocal increases in CD8 T cell responses in those who lacked anti-RBD antibodies. These studies provide insights into the role of B cells and humoral immunity in vaccine-induced T cell responses and shed light on the immune mechanisms that accompany aCD20 therapy based on differential responses to vaccination.

## Results

### Impact of aCD20 therapy on mRNA vaccine-induced antibody responses

**Fig. 1:**
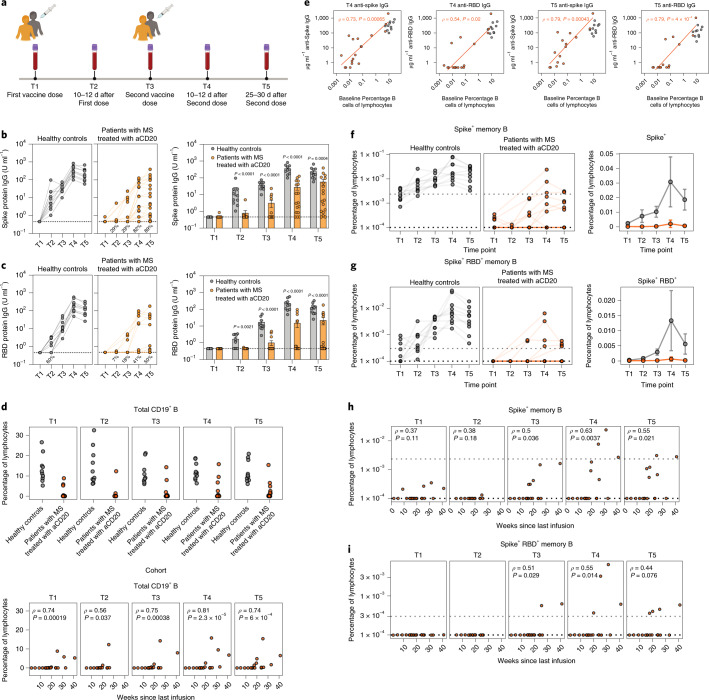
Decreased humoral responses after SARS-CoV-2 mRNA vaccination in patients with MS treated with aCD20. **a**, Longitudinal study design, vaccine administration scheme and time points collected after SARS-CoV-2 mRNA vaccination for healthy controls and patients with MS treated with aCD20. **b**,**c**, Anti-spike IgG (**b**) and anti-RBD IgG (**c**) for all time points collected (T1–T5) were measured in healthy controls and patients with MS treated with aCD20. Statistical analysis was performed using an unpaired, two-tailed, nonparametric Wilcoxon test. The bar plots represent the mean ± s.e.m. **d**, Top: Frequency of CD19^+^ B cells as a percentage of total lymphocytes in healthy controls and patients with MS treated with aCD20. Bottom: Correlation between the frequency of total CD19^+^ B cells and weeks since last aCD20 infusion. Correlations were calculated using nonparametric Spearman rank correlation. **e**, Correlations between the frequency of baseline (T1) percentage of B cells of all lymphocytes and levels of anti-spike IgG or anti-RBD IgG at T4 (left) and T5 (right) after vaccination. Only patients with MS treated with aCD20 were considered for the correlations, with healthy controls as a visual reference. Associations were calculated using Spearman rank correlation and are shown with Pearson trend lines for visualization. **f**,**g**, Frequency of spike^+^ (**f**) and spike^+^RBD^+^ (**g**) memory B cells over time in vaccinated individuals. Data are represented as the frequency of antigen-specific cells in the total lymphocyte compartment (left: individual points, log scale; right, mean with 95% CIs, linear scale). **h**,**i**, Correlation between the frequency of spike^+^ (**h**) and spike^+^RBD^+^ (**i**) memory B cells and weeks since last infusion of aCD20. Correlations were calculated using nonparametric Spearman rank correlation. Gray, healthy controls (*n* = 10); orange: patients with MS treated with aCD20 (*n* = 20).

**Extended Data Fig. 1:**
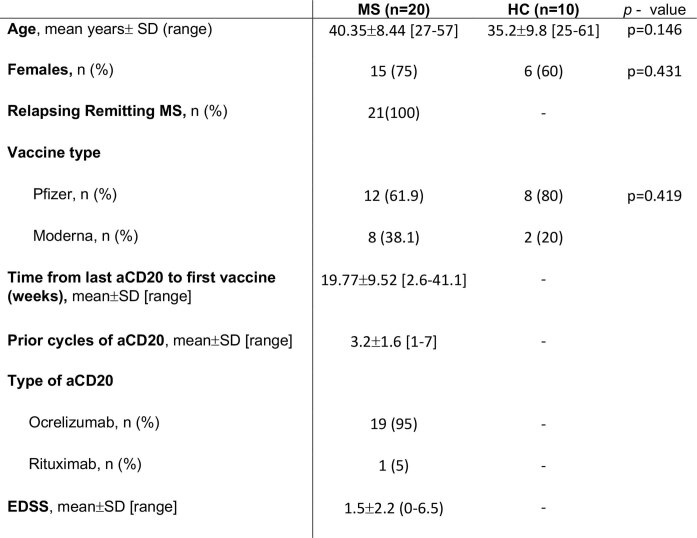
Clinical characteristics of patients with MS treated with aCD20 and healthy controls. Clinical characteristics and demographics of the HC and MS-aCD20 cohorts.

To examine the effect of aCD20 therapy on responses to SARS-CoV-2 mRNA vaccination, we recruited 20 patients with MS treated with aCD20 monotherapy and compared their vaccine-induced immune responses to 10 healthy controls (Extended Data Fig. 1). All patients with MS and healthy controls had no previous clinical signs or symptoms of COVID-19. Plasma and peripheral blood mononuclear cells (PBMCs) were analyzed at five time points before and after vaccination (Fig. 1a).

**Extended Data Fig. 2:**
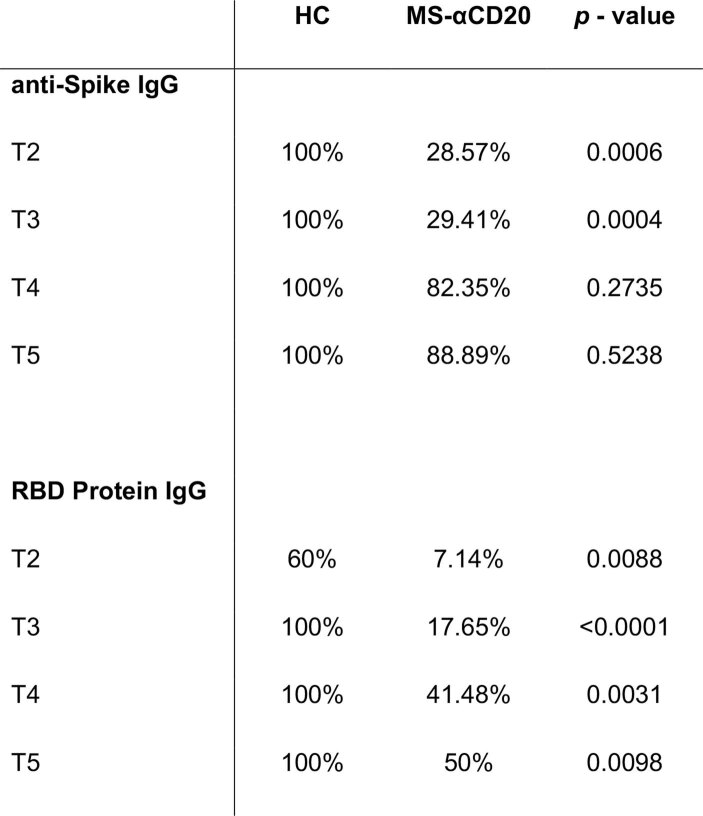
Spike and RBD serology positivity rates for patients with MS treated with aCD20 and healthy controls after SARS-CoV-2 mRNA vaccination. Anti-spike and anti-RBD IgG serological positivity rates in HC (n = 10) and MS-aCD20 patients (n = 20) across different time points. Two-sided Fisher’s exact test was calculated for each timepoint.

**Extended Data Fig. 3:**
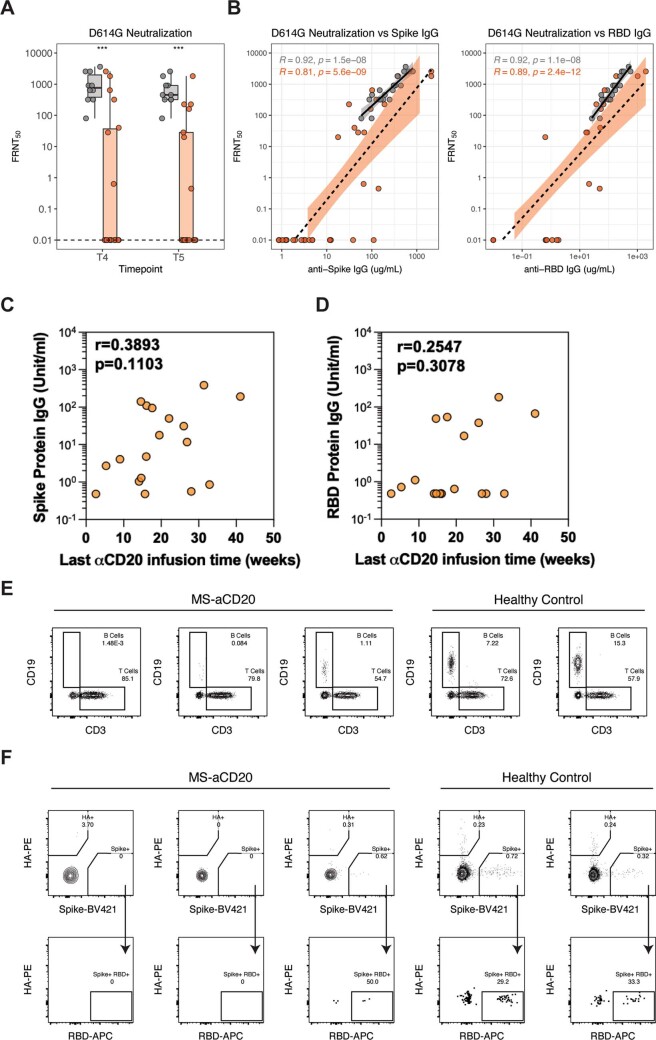
Neutralization data, correlation of serologies with last aCD20 infusion, and B cell responses of patients with MS treated with aCD20 and healthy controls after SARS-CoV-2 mRNA vaccination. **a)** Tukey boxplots (median, Q1 and Q3 quartiles) of FRNT_50_ values assayed against pseudotyped virus expressing SARS-CoV-2 D614G spike protein using serum samples from HCs (grey, n = 10) and MS-aCD20 patients (orange, n = 16) at timepoints T4 and T5 that were positive for anti-spike IgG; unpaired Wilcoxon test *P* values are shown. **b)** Spearman correlation analysis of anti-spike (left) and anti-RBD (right) IgG against D614G neutralization titers (HCs: grey, n = 10; MS-aCD20 patients, orange, n = 16). **c-d)** Spearman correlation analysis between the weeks elapsed since last aCD20 infusion administration and anti-spike IgG **(c)** or anti-RBD IgG **(d)** at T5 for MS-aCD20 patients (n = 20). **e)** Gating strategy and representative plots for flow cytometric analysis of total B cells. **f)** Gating strategy and representative plots for flow cytometric analysis of SARS-CoV-2-specific memory B cells. Cells were stained with fluorescently labeled SARS-CoV-2 full-length spike protein, SARS-CoV-2 spike receptor binding domain (RBD), and influenza hemagglutinin (HA). Spike^+^ HA^−^cells were subsequently analyzed for binding to RBD.

All healthy controls generated both anti-spike and anti-RBD IgG after the first dose of mRNA vaccine and antibody increased after the second dose (Fig. 1b,c), as reported^42^. By contrast, responses were more variable in patients with MS treated with aCD20, with 89% developing detectable anti-spike IgG and only 50% mounting detectable anti-RBD IgG responses by T5 (Fig. 1b,c and Extended Data Fig. 2). Among those patients with MS treated with aCD20 with detectable IgG, the magnitude was generally lower and the kinetics of the IgG response delayed compared to healthy controls. Moreover, the generation of neutralizing antibody by T4 and T5 was significantly reduced in the MS-aCD20 group (Extended Data Fig. 3a). Neutralizing and binding antibody titers for spike and RBD were correlated for both patients and healthy controls (Extended Data Fig. 3b). These findings extend previous observations^24,25^ that antibody responses to SARS-CoV-2 mRNA vaccine are attenuated in patients with MS on aCD20 therapy.

Because a major reason for the altered antibody responses in patients with MS treated with aCD20 was likely to be depletion of B cells, we considered whether the heterogeneity in antibody responses (Fig. 1b,c) was related to the duration between vaccination and the last aCD20 infusion. There were trends toward increased serologic responses to both spike (Extended Data Fig. 3c) and RBD (Extended Data Fig. 3d) as the duration from the last aCD20 infusion increased. To further test this idea, we quantified CD19^+^ B cell numbers in circulation (Extended Data Fig. 3e). Although most patients with MS treated with aCD20 had no detectable B cells, small circulating B cell populations were observed in some patients and there was a clear relationship between time since last aCD20 infusion and the extent of B cell reconstitution (Fig. 1d). Patients with MS treated with aCD20 with higher percentages of circulating B cells before the vaccine (T1) had more robust anti-spike and anti-RBD IgG responses at T4 and T5 (Fig. 1e), demonstrating a correlation between mRNA vaccine antibody responses and the extent of B cell reconstitution at the time of vaccination. The small number of patients with MS treated with aCD20 who had circulating B cell frequencies comparable to healthy controls achieved equivalent antibody titers after vaccination (Fig. 1e), which suggests that B cells repopulating the periphery after aCD20 infusion are functionally competent. Thus, when the circulating B cell pool is repopulated with increased time since last aCD20 administration, vaccine-induced antibody responses approached those observed in healthy controls.

### aCD20 effects on vaccine-induced antigen-specific memory B cells

We next used a spike and RBD B cell probe strategy^42^ to define the magnitude and kinetics of the memory B cell response in patients with MS treated with aCD20 after SARS-CoV-2 mRNA vaccination (Methods). Although circulating memory B cells specific for both spike (Extended Data Fig. 3f and Fig. 1f) and RBD (Extended Data Fig. 3f and Fig. 1g) were readily induced in all healthy controls, spike-specific memory B cells were detected in only a subset of patients with MS treated with aCD20, where their frequencies were also substantially diminished (Fig. 1f) at all time points (Supplementary Table 1). Similarly, only a minority of patients with MS treated with aCD20 generated detectable RBD-specific memory B cells (Fig. 1g and Supplementary Table 1). Finally, there was a strong correlation between detection of antigen-specific memory B cells and longer duration since the last aCD20 treatment (Fig. 1h,i). There were substantially more patients with detectable antibody responses (88.9%) than patients with detectable circulating memory B cells (30%) to the spike antigen, perhaps suggesting a role for repopulation of B cells in lymphoid tissues before the blood. Overall, however, these data indicate that memory B cell responses after SARS-CoV-2 mRNA vaccination were compromised in patients with MS treated with aCD20 compared with healthy controls especially in patients who were immunized in closer proximity to their last aCD20 infusion.

### aCD20 impact on vaccine-induced CD4 T cell responses

**Extended Data Fig. 4:**
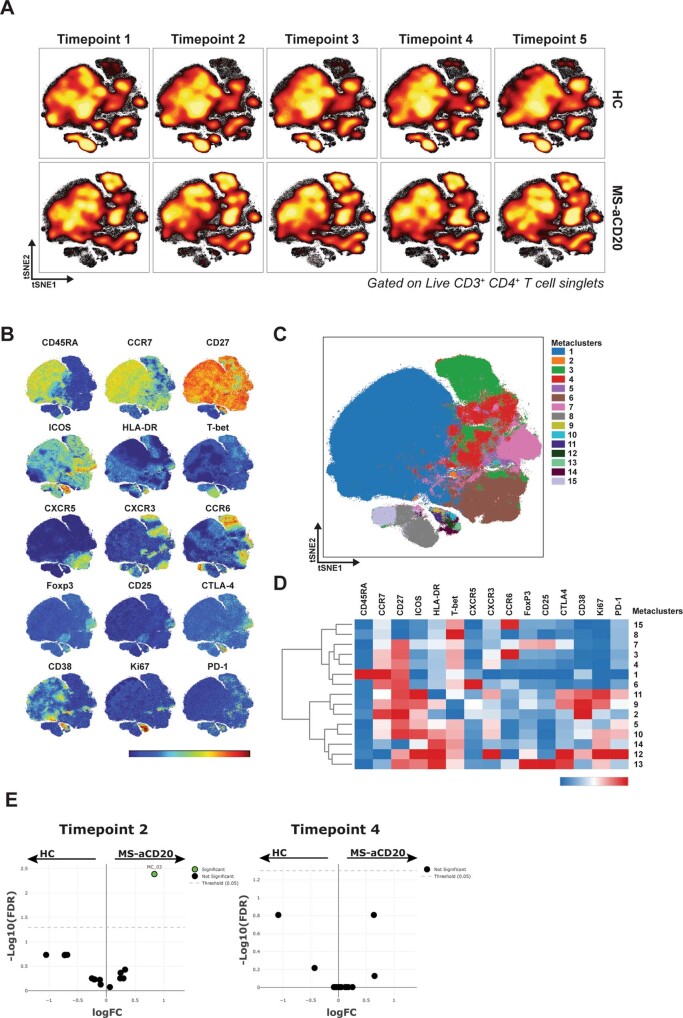
CD4 T cell responses of patients with MS treated with aCD20 and healthy controls after SARS-CoV-2 mRNA vaccination. **a)** Opt-SNE projections of concatenated cytometry data for total CD3^+^ CD4^+^ T cells for each timepoint and group combination are shown. **b)** Surface expression intensity of the indicated markers projected on the opt-SNE 2D-map generated with all samples in **(a)** (color scale: MFI expression of each individual marker in a log scale). **c)** FlowSOM metaclusters were created using total CD3^+^ CD4^+^ T cells concatenated from all samples and projected to the opt-SNE map. **d)** Surface expression intensity heatmap of the markers indicated for each of the 15 FlowSOM metaclusters in **(c)** (color scale: row-adjusted z-score expression for each individual marker). **e)** Volcano plots of the logFC (log fold change) of the abundance between the HC and MS-aCD20 groups for each of the 15 metaclusters indicated in **(c)** and the -log10 value of the false discovery rate (FDR) for timepoints 2 and 4. HCs (n = 10) and MS-aCD20 patients (n = 20).

The impact of aCD20 treatment on T cell responses to SARS-CoV-2 mRNA vaccination is unclear. To examine this question, we implemented high-dimensional flow cytometry analysis of circulating T cell populations after SARS-CoV-2 mRNA vaccination, using optimized *t*-distributed stochastic neighbor embedding (opt-SNE) dimensionality reduction followed by FlowSOM clustering (Supplementary Fig. 1 and Extended Data Fig. 4). Examining the total CD4^+^ T cell landscape over time revealed dynamic changes after mRNA vaccination (Extended Data Fig. 4a). The total landscape was mapped with key markers (Extended Data Fig. 4b) and metaclusters corresponding to distinct subpopulations of CD4 T cells were defined (Extended Data Fig. 4c,d). A group of small metaclusters (metaclusters 9–14) was identified that expanded after the first vaccine dose in healthy controls and expressed high Ki67, CD38, inducible costimulator (ICOS) and human leukocyte antigen-DR isotype (HLA-DR), consistent with vaccine-induced activated T cells. These metaclusters showed less dynamic change in the MS-aCD20 group with more subtle induction at T2 and T4. No differences were observed in the abundance of these metaclusters between the MS-aCD20 and healthy control groups at either T2 or T4 (Extended Data Fig. 4e). We next wanted to gain deeper insights into the CD4 T cell subpopulations induced by vaccination in MS-aCD20 patients compared with healthy controls.

**Fig. 2:**
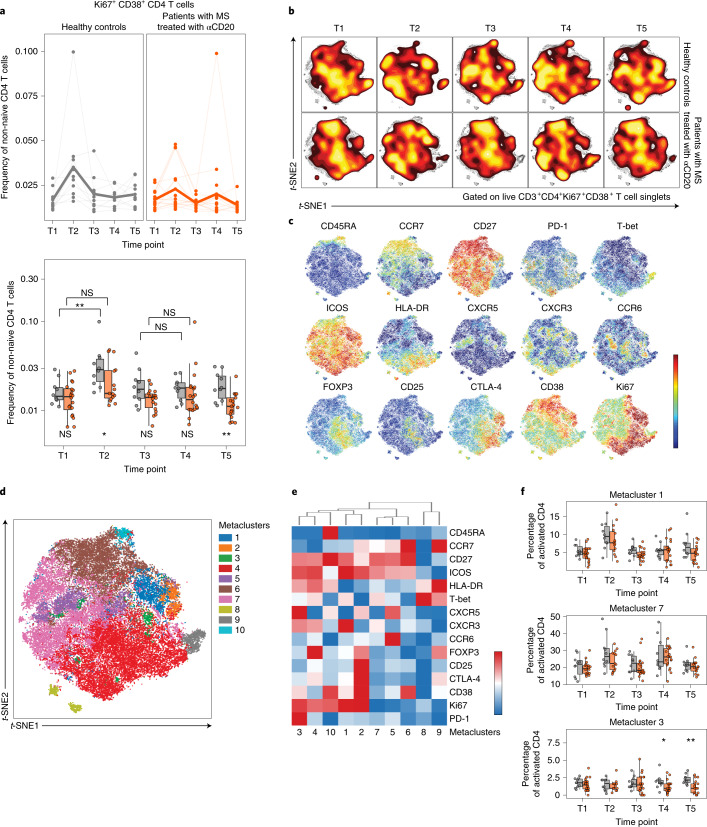
SARS-CoV-2 mRNA vaccination results in altered CD4 T cell activation in patients with MS treated with aCD20. **a**, The frequency of activated Ki67^+^CD38^+^ CD4 T cells of total non-naive CD4 T cells. Top: Individuals (points) and the mean (thicker line) are shown for each group. Bottom: Tukey box plots (median, Q1 and Q3 quartiles) for each time point and group are depicted. An unpaired, two-tailed Wilcoxon test was used to compare the two groups at each time point (shown under the box plots) or the groups between the time points indicated (shown above the box plots). NS, not significant. **b**, Opt-SNE projections of concatenated cytometry data for activated Ki67^+^CD38^+^ CD4 T cells for each time point and group combination are shown. **c**, Surface expression intensity of the indicated markers projected on the opt-SNE two-dimensional (2D) map generated with all samples in **b** (color scale: mean fluorescence intensity (MFI) expression of each individual marker in a log scale). **d**, FlowSOM metaclusters were created using activated Ki67^+^CD38^+^ CD4 T cells concatenated from all samples and projected to the opt-SNE map. **e**, Surface expression intensity heatmap of the markers indicated for each of the ten FlowSOM metaclusters in **d** (color scale: row-adjusted *z*-score expression for each individual marker). **f**, Abundance of metaclusters 1, 7 and 3 as the percentage of activated Ki67^+^CD38^+^ CD4 T cells. Unpaired, two-tailed Wilcoxon test *P* values are shown when *P* < 0.05 between groups. Gray, healthy controls (*n* = 10); orange, patients with MS treated with aCD20 (*n* = 20). *P < 0.05, **P < 0.01.

**Extended Data Fig. 5:**
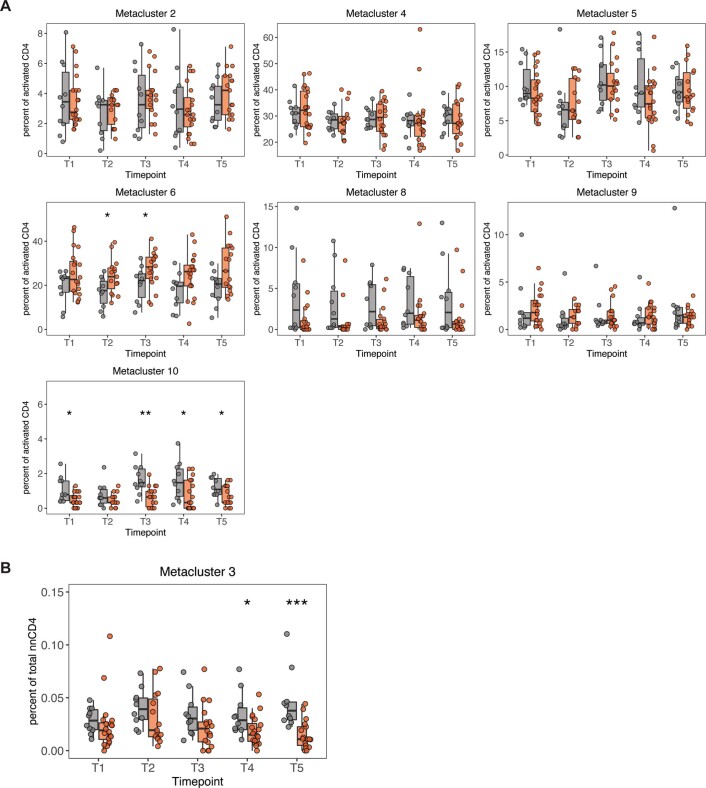
FlowSOM metaclusters of activated CD4 T cells for patients with MS treated with aCD20 and healthy controls after SARS-CoV-2 mRNA vaccination. **a)** The abundance of metaclusters 2, 4, 5, 6, 8, 9 and 10 as percentage of activated Ki67^+^CD38^+^ CD4 T cells is shown for HC (grey) and MS-aCD20 (orange) groups; Tukey boxplots (median, Q1 and Q3 quartiles) are shown; unpaired, two-tailed Wilcoxon test *p* values are depicted when *P* < 0.05 between groups. **b)** The abundance of metacluster 3 as percentage of total non-naive CD4 T cells is shown for HC (grey) and MS-aCD20 (orange) groups; Tukey boxplots (median, Q1 and Q3 quartiles) are shown; unpaired, two-tailed Wilcoxon test *P* values are depicted when *P* < 0.05 between groups. HCs (n = 10) and MS-aCD20 patients (n = 20). For *P* values: * indicates *P* < 0.05, ** indicates *P* < 0.01, *** indicates *P* < 0.001.

Vaccination in humans induces Ki67^+^CD38^+^ CD4 and CD8 T cells approximately 1–2 weeks after Se immunization; this activated, proliferating subset contains antigen-specific T cells^43–46^. Consistent with previous reports^47^, a population of Ki67^+^CD38^+^ CD4 T cells was induced after the first vaccine dose in healthy controls, peaking at T2 and then returning to baseline (Fig. 2a and Supplementary Fig. 1). Patients with MS treated with aCD20 had similar frequencies of activated CD4 T cells at baseline. However, their Ki67^+^CD38^+^ CD4 T cells were less induced after vaccination compared to healthy controls at T2, displayed no increase after the second dose and remained lower than healthy controls through T5 (Fig. 2a). Comparison of these activated Ki67^+^CD38^+^ CD4 T cells revealed landscape differences independent of vaccination or time point in patients with MS treated with aCD20 versus healthy controls (Fig. 2b). However, there were also clear patterns of vaccine-induced change in subpopulations of Ki67^+^CD38^+^ CD4 T cells. There were areas of more intense Ki67 or CD38 expression, as well as areas of cells that expressed FOXP3, CTLA-4, CXCR5, CXCR3, CCR6, T-bet and other activation markers corresponding to distinct subpopulations of activated CD4 T cells (Fig. 2c–e and Extended Data Fig. 5a). Additional metaclusters were identified with clear enrichment after vaccination. Specifically, metacluster 1 increased at T2 and metacluster 7 increased at both T2 and T4 (Fig. 2f). Metacluster 1 was composed of highly activated Ki67^++^ICOS^++^CXCR3^+^T-bet^mid^ CD4 T cells of the central memory (T_CM_)/type 1 effector memory (T_EM1_) phenotype (T_CM_/T_EM1_ T_H_1 cells). Metacluster 7 represented CCR6^+^T-bet^−^ or CXCR3^+^T-bet^mid^ T_CM_/T_EM1_ CD4 T cells with high ICOS (T_CM_/T_EM1_ T_H_17- and T_H_1-like cells). The dynamic changes in these two metaclusters after vaccination were similar between the MS-aCD20 and healthy control groups (Fig. 2f). We next sought to understand the response of circulating T_FH_ cells given the role of T_FH_ cells in supporting antigen-specific B cell responses. Metacluster 3 was an activated (CD38^+^ICOS^+^HLA-DR^+^), proliferating (Ki67^+^) subpopulation with high expression of CXCR5 and PD-1 (Fig. 2e), corresponding to activated circulating T_FH_ cells. This metacluster was similarly induced after the first vaccine dose for both patients with MS treated with aCD20 and healthy controls (Fig. 2f and Extended Data Fig. 5b). However, after the second vaccine dose and through T5, metacluster 3 decreased in proportion (Fig. 2f) and contracted (Extended Data Fig. 5b) in patients with MS treated with aCD20 compared to healthy controls. Thus, this analysis identified subpopulations of CD4 T cells that responded similarly to vaccination when comparing patients with MS treated with aCD20 to healthy controls (for example, subsets of activated T_H_1 cells) as well as circulating T_FH_ cells that had similar initial induction in the two cohorts but poor maintenance in patients with MS treated with aCD20.

**Fig. 3:**
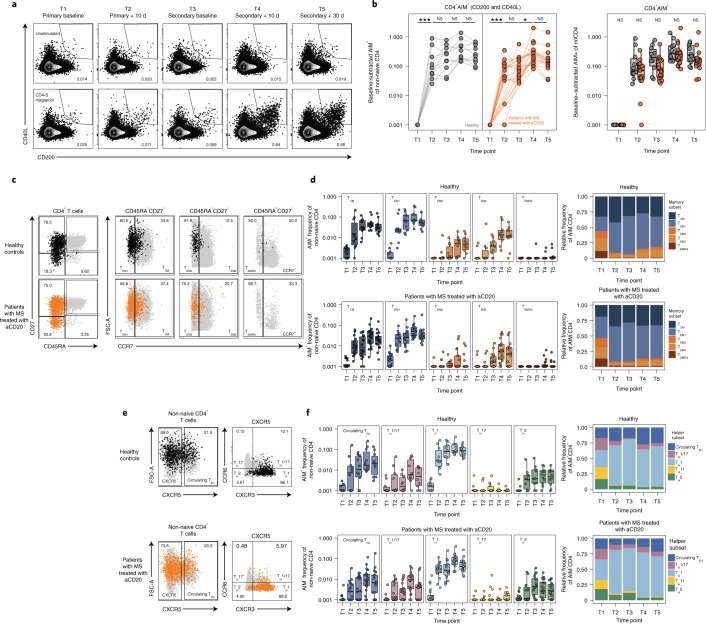
Vaccine-specific CD4 T cell responses are comparable between patients with MS treated with aCD20 and healthy controls. **a**, Representative flow cytometry plots for the quantification of AIM^+^CD4 T cells. The numbers represent the percentage of total non-naive CD4 T cells that are AIM^+^. Top row: Unstimulated. Bottom row: Stimulated with the CD4-S megapool. **b**, Summary data of AIM^+^CD4 T cell frequency after vaccination. The values represent the background-subtracted frequency of AIM^+^ non-naive CD4 T cells above paired background-subtracted baseline frequencies. The lines connect individual donors sampled longitudinally. Statistics were calculated using an unpaired, two-tailed Wilcoxon test. Gray, healthy controls (*n* = 10); orange, patients with MS treated with aCD20 (*n* = 20). **c**, Representative plots demonstrating the identification of the indicated memory T cell subsets from AIM^+^CD4 T cells. The black or orange events depict AIM^+^ cells from healthy controls or patients with MS treated with aCD20, respectively. The gray events depict the total CD4 T cells from the same donor. The numbers indicate the frequency of AIM^+^ cells within each gate. **d**, Frequency of memory T cell subsets in AIM^+^CD4 T cells. Top: Healthy controls. Bottom: Patients with MS treated with aCD20. Left: Background-subtracted percentage of non-naive T cells that are AIM^+^ cells of each subset. Right: Relative frequency of each memory T cell subset in the background-subtracted AIM^+^ population. T_CM_ = CD45RA^−^CD27^+^CCR7^+^; T_EM1_ = CD45RA^−^CD27^+^CCR7^−^; T_EM2_ = CD45RA^−^CD27^−^CCR7^+^; T_EM3_ = CD45RA^−^CD27^−^CCR7^−^, T_EMRA_ = CD45RA^+^CD27^−^CCR7^−^. **e**, Representative flow cytometry plots depicting the gating of AIM^+^CD4 T cells to identify the indicated helper subsets as in **c**. **f**, Frequency of helper T cell subsets in AIM^+^CD4 T cells as in **d**. Circulating T_FH_ = CXCR5^+^ of non-naive CD4 T cells; T_H_1 = CXCR5^−^CXCR3^+^CCR6^−^; T_H_2 = CXCR5^−^CXCR3^−^CCR6^−^; T_H_17 = CXCR5^−^CXCR3^−^CCR6^+^; T_H_1/17 = CXCR5^−^CXCR3^+^CCR6^+^. Healthy controls (*n* = 10); patients with MS treated with aCD20 (*n* = 20). *P < 0.05, **P < 0.01, ***P < 0.001.

**Extended Data Fig. 6:**
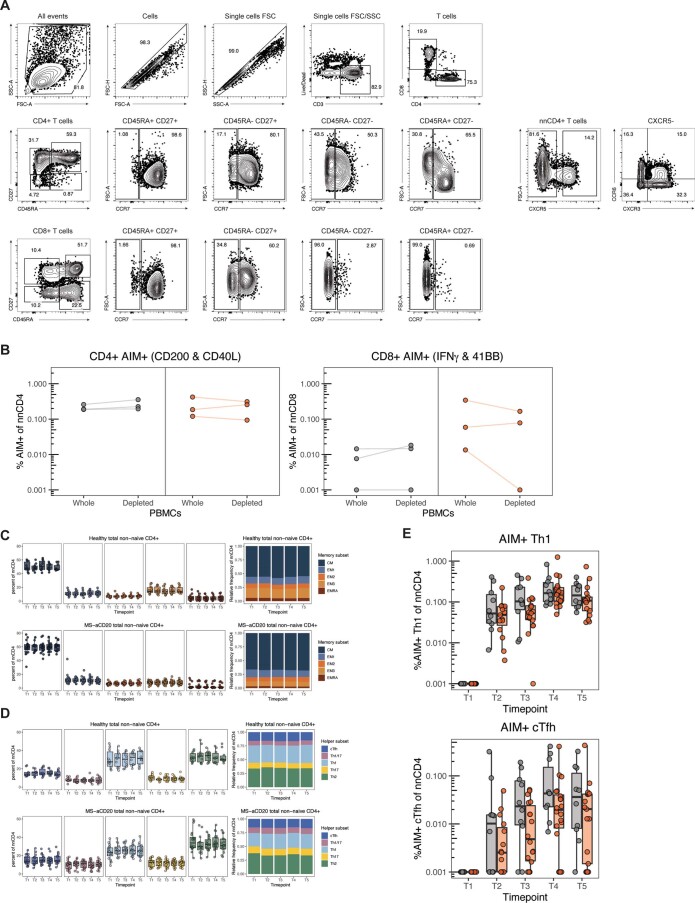
AIM^+^ CD4 T cell gating strategy and total CD4 T cell memory and helper subsets in patients with MS treated with aCD20 and healthy controls after SARS-CoV-2 mRNA vaccination. **a)** Gating strategy for identifying T cell subsets. **b)** Timepoint 4 AIM^+^ CD4 T cell (left) and CD8 T cell (right) frequency in whole PBMCs or PBMCs depleted of B cells by magnetic separation. Values represent the background-subtracted frequency of AIM^+^ non-naive CD4 T cells above paired baseline frequencies. Lines connect paired samples from individual donors. Grey indicates HC (n = 3); orange indicates MS-aCD20 patients (n = 3). **c)** Frequency of memory CD4 T cell subsets in total non-naive CD4 T cells. Left panels depict the percent of total non-naive T cells that are in each subset. Right panels depict the relative frequency of each memory T cell subset in the total non-naive population. CM = CD45RA^−^CD27^+^ CCR7^+^, EM1 = CD45RA^-^CD27^+^ CCR7^−^, EM2 = CD45RA^−^CD27^−^CCR7^+^, EM3 = CD45RA^−^CD27^−^CCR7^−^, EMRA = CD45RA^+^ CD27^−^CCR7^−^. **d)** Frequency of T helper subsets in total non-naive CD4 T cells. Left panel depicts the percent of total non-naive T cells that are helper T cells in each subset. Right panel depicts the relative frequency of each helper T cell subset in the total non-naive population. cTfh = CXCR5^+^, Th1 = CXCR5^−^CXCR3^+^ CCR6^−^, Th2 = CXCR5^−^CXCR3^−^CCR6^−^, Th17 = CXCR5^−^CXCR3^−^CCR6^+^, Th1/17 = CXCR5^−^CXCR3^+^ CCR6^+^. **e**) Summary data of AIM^+^ frequencies of the indicated T cell populations following vaccination. Values represent the background-subtracted frequency of AIM^+^ non-naive T cells above paired baseline frequencies. Tukey boxplots (median, Q1 and Q3 quartiles) are shown; unpaired, two-tailed Wilcoxon test *P* values are depicted when *P* < 0.05 between the HC (grey, n = 10) and MS-aCD20 (orange, n = 20) groups.

To examine bona fide antigen-specific CD4 T cell responses, we performed spike peptide-dependent activation-induced marker (AIM) assays (Methods). AIM^+^CD4^+^ T cells were defined by coexpression of CD200 and CD40L (Fig. 3a and Extended Data Fig. 6a). The absence of B cells during the AIM peptide stimulation assay did not impact this assay (Extended Data Fig. 6b). After the first dose of the SARS-CoV-2 mRNA vaccine, AIM^+^CD4 T cells were robustly increased in MS-aCD20 and healthy controls, indicating efficient CD4 T cell priming (Fig. 3b). Healthy controls retained high AIM^+^CD4 T cell frequencies at all subsequent time points with a trend toward an additional increase after the second vaccine dose (Fig. 3b), which is consistent with our previous studies^47^. AIM^+^CD4 T cell responses in patients with MS treated with aCD20 were comparable to healthy controls at all time points examined (Fig. 3b). To further assess memory T cell subsets, we subdivided AIM^+^CD4 T cells into T_CM_, three different subpopulations of effector memory T cells (T_EM1_, T_EM2_, T_EM3_) and T_EM_ cells reexpressing CD45RA (T_EMRA_) (Fig. 3c and Extended Data Fig. 6c). There were no major differences in the distribution of AIM^+^CD4 T cells among these memory T cell subsets between patients with MS treated with aCD20 and healthy controls, with most AIM^+^CD4 T cells mapping to the T_CM_ and T_EM1_ subsets (Fig. 3d) in both groups. Similarly, we used CXCR5, CXCR3 and CCR6 (Fig. 3e and Extended Data Fig. 6d) to examine CD4 T_H_ cell subsets. Although the distribution was largely similar between the cohorts, there was a trend toward a lower frequency of circulating T_FH_ cells among the total AIM^+^-responding CD4 T cells in the MS-aCD20 group (Fig. 3f and Extended Data Fig. 6e). Thus, these data indicate that patients with MS treated with aCD20 were capable of generating robust antigen-specific CD4 T cell responses to both vaccine doses despite attenuated antibody responses.

### aCD20 impact on vaccine-induced CD8 T cell responses

**Fig. 4:**
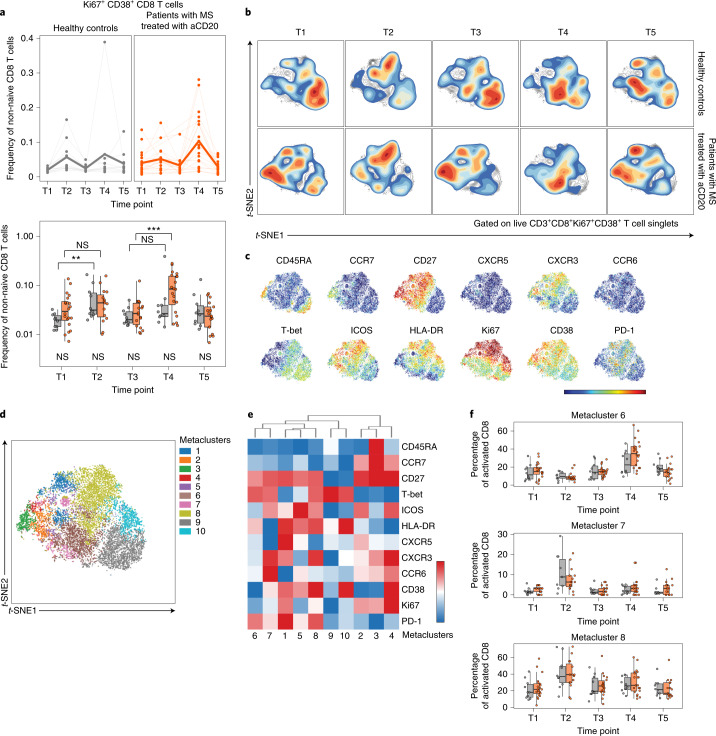
SARS-CoV-2 mRNA vaccination results in robust CD8 T cell activation in healthy controls and patients with MS treated with aCD20. **a**, Frequency of activated Ki67^+^CD38^+^ CD8 T cells of total non-naive CD8 T cells. Top: Individuals (points) and the mean (thicker line) are shown for each group. Bottom: Tukey box plots (median, Q1 and Q3 quartiles) for each time point and group are depicted. An unpaired, two-tailed Wilcoxon test was used to compare the two groups at each time point (shown under the box plots) or the groups between time points indicated (shown above the box plots). **b**, The opt-SNE projections of concatenated cytometry data for activated Ki67^+^CD38^+^ CD8 T cells for each time point and group combination are shown. **c**, Surface expression intensity of the indicated markers projected on the opt-SNE 2D map generated with all samples in **b** (color scale: MFI expression of each individual marker in a log scale). **d**, FlowSOM metaclusters were created using activated Ki67^+^CD38^+^ CD8 T cells concatenated from all samples and projected onto the opt-SNE map. **e**, Surface expression intensity heatmap of the markers indicated for each of the ten FlowSOM metaclusters in **d** (color scale: row-adjusted *z*-score expression for each individual marker). **f**, The abundance of metaclusters 6, 7 and 8 as the percentage of activated Ki67^+^CD38^+^ CD8 T cells is shown for healthy controls and patients with MS treated with aCD20. Unpaired, two-tailed Wilcoxon test *P* values are shown when *P* < 0.05 between groups. Gray, healthy controls (*n* = 10); orange: patients with MS treated with aCD20 (*n* = 20). *P < 0.05, **P < 0.01, ***P < 0.001.

**Extended Data Fig. 7:**
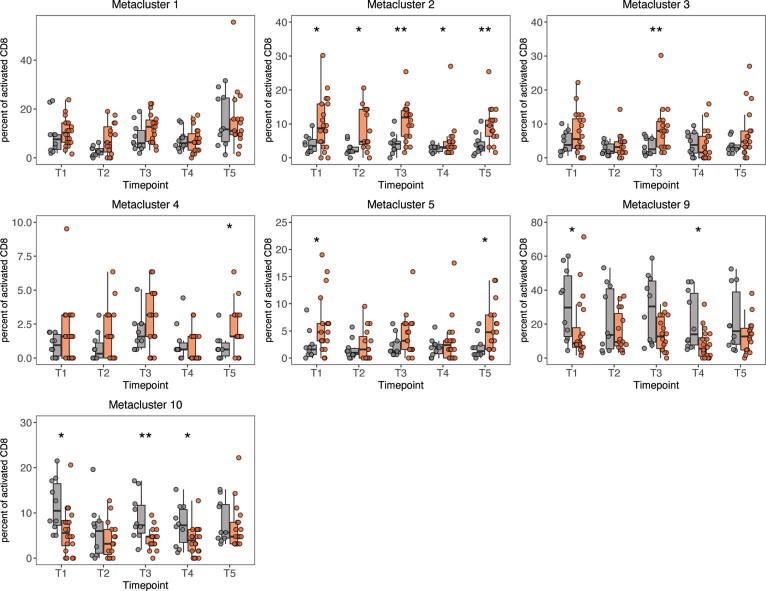
FlowSOM metaclusters of activated CD8 T cells for patients with MS treated with aCD20 and healthy controls after SARS-CoV-2 mRNA vaccination. The abundance of metaclusters 1, 2, 3, 4, 5, 9 and 10 as percentage of activated Ki67^+^ CD38^+^ CD8 T cells is shown for HC (grey, n = 10) and MS-aCD20 (orange, n = 20) groups; unpaired, two-tailed Wilcoxon test p values are shown when P < 0.05 between groups. For *P* values: * indicates *P* < 0.05, ** indicates *P* < 0.01.

We next examined CD8 T cell responses after vaccination in patients with MS treated with aCD20 and healthy controls. We first assessed activated Ki67^+^CD38^+^CD8 T cells (Fig. 4) as above for CD4 T cells. Activated CD8 T cells moderately expanded after the first vaccine dose in both cohorts, although the magnitude of increase was more robust for healthy controls (Fig. 4a), possibly due to higher pre vaccination (T1) CD8 T cell activation in the MS-aCD20 group. However, patients with MS treated with aCD20 generated a considerably stronger response to the second vaccine dose than the healthy control group. We next applied the metaclustering approach described above for CD4 T cells to interrogate the differentiation state of the vaccine-responding activated Ki67^+^CD38^+^CD8 T cells (Fig. 4b–f and Extended Data Figure 7). The opt-SNE landscape map of activated CD8 T cells revealed differences between patients with MS treated with aCD20 and healthy controls before vaccination, including an abundance of CD27^+^ICOS^+^CD38^+^CD8 T cells largely lacking T-bet in patients with MS treated with aCD20 in contrast to CD27^−^T-bet^+^ CD8 T cells in healthy controls (Fig. 4b). However, the activated CD8 T cell populations in both patients and healthy controls reoriented after each vaccine dose, such that they occupied a similar opt-SNE space (Fig. 4b). Metaclusters defined these vaccine-induced changes (Fig. 4d–f and Extended Data Figure 7). Specifically, metacluster 7 and 8 were the main vaccine-responding CD8 T cell populations in both groups after the first vaccine dose (Fig. 4b,f), representing T_EM1_ CD8 T cells with high T-bet, ICOS and CXCR3. Metacluster 8 expressed high HLA-DR, CD38 and PD-1 and comprised a larger fraction of the activated CD8 T cell pool at T2 compared to metacluster 7 (Fig. 4e,f). Metacluster 6 was the predominant population enriched after the second vaccine dose in both groups (Fig. 4b,f). Like metaclusters 7 and 8, metacluster 6 was a T_EM1_ subset that expressed T-bet. However, metacluster 6 expressed intermediate or high HLA-DR and PD-1 but lower ICOS, CXCR3 and CD38 compared to metaclusters 7 and 8 (Fig. 4e). Thus, these high-dimensional cytometry data indicated that vaccine-induced activated CD8 T cell responses were more robust in patients with MS treated with aCD20 compared to healthy controls after the second vaccine dose. Moreover, the predominant responding CD8 T cell subsets were T-bet^+^ T_EM1_ cells with variable levels of activation and CXCR3 expression.

**Fig. 5:**
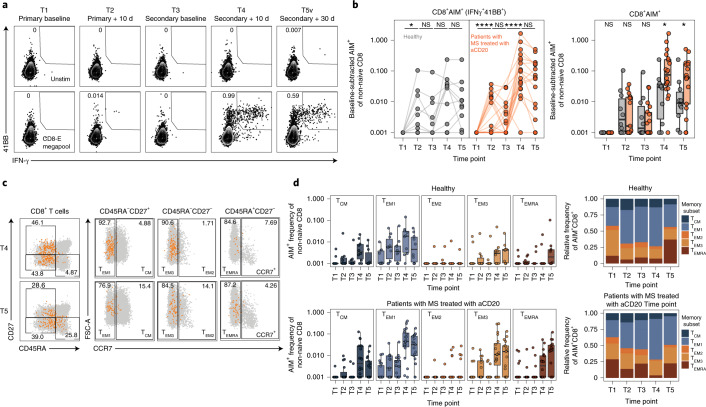
Enhanced antigen-specific CD8 T cell responses after mRNA vaccination in patients with MS treated with aCD20. **a**, Representative flow cytometry plots for quantifying AIM^+^CD8 T cells. The numbers represent the percentage of total non-naive CD8 T cells that are AIM^+^. Top: Unstimulated. Bottom: Stimulated with the CD8-E megapool. **b**, Summary data of AIM^+^CD8 T cell frequency after vaccination. The values represent the background-subtracted frequency of AIM^+^ non-naive CD8 T cells above paired background-subtracted baseline frequencies. The lines connect individual donors sampled longitudinally. Statistics were calculated using an unpaired, two-tailed Wilcoxon test. Gray, healthy controls; orange: patients with MS treated with aCD20. **c**, Representative flow cytometry plots depicting the identification of the indicated memory T cell subsets from AIM^+^CD8 T cells at T4 and T5. The orange events depict AIM^+^ cells from patients with MS treated with aCD20. The gray events depict the total CD8 T cells from the same donor. The numbers indicate the frequency of AIM^+^ cells within each gate. **d**, Frequency of memory T cell subsets in AIM^+^CD8 T cells. Top: Healthy controls. Bottom: Patients with MS treated with aCD20. Left: Background-subtracted percentage of non-naive T cells that are AIM^+^ cells of each subset. Right: Relative frequency of each memory T cell subset in the background-subtracted AIM^+^ population. T_CM_ = CD45RA^−^CD27^+^CCR7^+^; T_EM1_ = CD45RA^−^CD27^+^CCR7^−^; T_EM2_ = CD45RA^−^CD27^−^CCR7^+^; T_EM3_ = CD45RA^−^CD27^−^CCR7^−^; T_EMRA_ = CD45RA^+^CD27^−^CCR7^−^. Healthy controls (*n* = 10); patients with MS treated with aCD20 (*n* = 20). *P < 0.05, **P < 0.01, ***P < 0.001, ****P < 0.0001.

**Extended Data Fig. 8.**
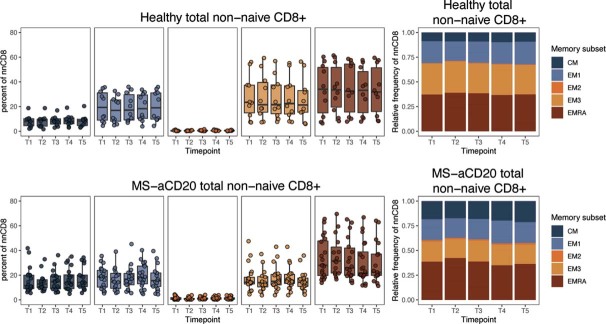
Memory CD8 T cell subsets of patients with MS treated with aCD20 and healthy controls after SARS-CoV-2 mRNA vaccination. Frequency of memory CD8 T cell subsets in total non-naive CD8 T cells. Left panels depict the percent of total non-naive T cells that are in each subset. Right panels depict the relative frequency of each memory T cell subset in the total non-naive population. CM = CD45RA^−^CD27^+^ CCR7^+^, EM1 = CD45RA^−^CD27^+^ CCR7^−^, EM2 = CD45RA^−^CD27^−^CCR7^+^, EM3 = CD45RA^−^CD27^−^CCR7^−^, EMRA = CD45RA^+^ CD27^−^CCR7^−^. Top panel: HCs (n = 10). Bottom panel: MS-aCD20 (n = 20).

**Extended Data Fig. 9:**
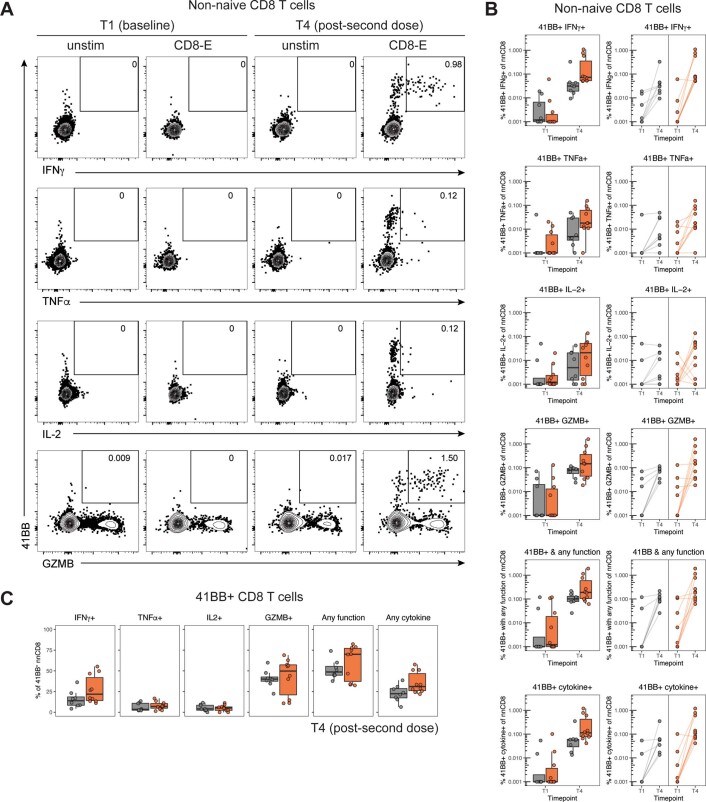
Cytokine and Granzyme B production of AIM^+^ CD8 T cells of patients with MS treated with aCD20 and healthy controls after SARS-CoV-2 mRNA vaccination. **a)** Representative flow cytometry plots for quantifying IFN-γ, TNF, IL-2 and Granzyme B expression in AIM^+^ CD8 T cells. Numbers represent the percentage of AIM^+^ non-naive CD8 T cells that co-express 41BB and the corresponding cytokine or Granzyme B. **b)** Tukey boxplots (median, Q1 and Q3 quartiles, left) and individual points (right) of the frequency of non-naive CD8 T cells that are 41BB^+^ and express the indicated cytokines and/or Granzyme B at timepoints T1 and T4 following vaccination. Lines connect individual donors sampled longitudinally. Grey indicates HCs (n = 8), orange indicates MS-aCD20 patients (n = 10). **c)** Tukey boxplots (median, Q1 and Q3 quartiles) of the percentage of 41BB^+^ non-naive CD8 T cells that express the indicated cytokines and/or Granzyme B at timepoint T4 following vaccination. Grey indicates HCs (n = 8), orange indicates MS-aCD20 patients (n = 10). Any function = at least one of IFN-γ, TNF, IL-2, and Granzyme B. Any cytokine = at least one of IFN-γ, TNF, and IL-2.

We next examined antigen-specific CD8 T cell responses after vaccination using spike-dependent AIM assays (Fig. 5). As reported^47^, AIM^+^CD8 T cell responses were detected in a subset of healthy controls after the first vaccine dose, with more individuals responding after the second vaccine dose (Fig. 5a,b). A similar pattern was observed in the patients with MS treated with aCD20. However, after the second vaccine dose (T4), a significantly greater expansion of antigen-specific CD8 T cells was noted in patients with MS treated with aCD20 compared to healthy controls, a difference that persisted at T5. This expansion was dominated by T_EM1_ cells (Fig. 5c,d) consistent with the observations above. Of note, both groups had equivalent frequencies of total memory subsets that were largely unchanged by vaccination (Extended Data Figure 8). To assess the functionality of vaccine-specific CD8 T cells, we evaluated the expression of interferon-γ (IFNγ), tumor necrosis factor (TNF), interleukin-2 (IL-2) and granzyme B in AIM^+^CD8 T cells at T4, the peak of the response (Extended Data Figure 9a–c). CD8 T cells at T4 were similarly capable of producing these effector molecules in the healthy control and MS-aCD20 groups whereas there was little granzyme B expression or antigen-specific cytokine production at T1 before vaccination. Taken together, these data demonstrate that although the overall distribution of memory CD8 T cell subsets was similar, SARS-CoV-2 mRNA vaccination induced a more robust antigen-specific CD8 T cell response in patients with MS treated with aCD20 compared to healthy controls, in particular after the second dose of the vaccine.

### MS-aCD20 subgroups with distinct vaccine-induced immune coordination

**Fig. 6:**
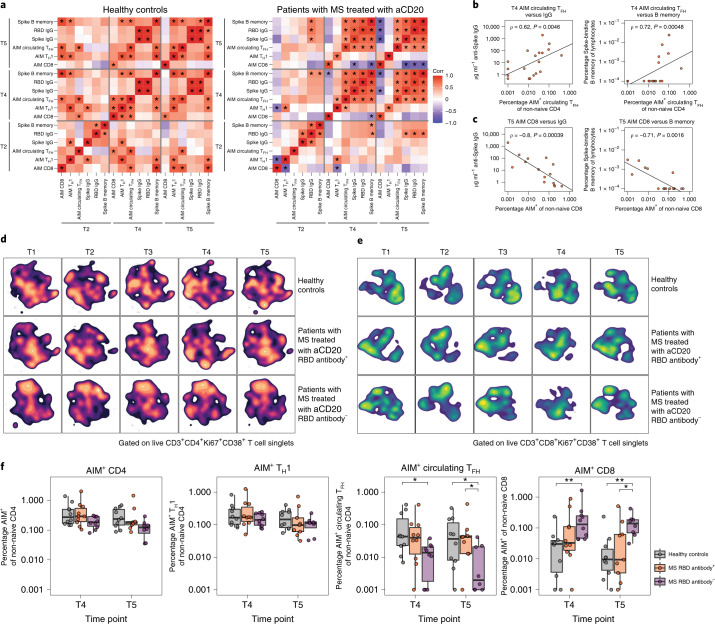
Patients with MS treated with aCD20 with no detectable anti-RBD IgG demonstrate the highest level of immune discoordination after SARS-CoV-2 mRNA vaccination. **a**, Correlations of antigen-specific features at T2, T4 and T5 in healthy controls (left, *n* = 10) and patients with MS treated with aCD20 (right, *n* = 20). Associations were calculated using Spearman rank correlation; *P < 0.05. **b**,**c**, Correlations between the frequency of T4 AIM^+^ circulating T_FH_ cells and T4 anti-spike IgG (left) or spike-specific memory B cells (right) (**b**) and between the frequency of T5 AIM^+^CD8 T cells and T5 anti-spike IgG (left) or spike-specific memory B cells (right) (**c**). Only patients receiving aCD20 therapy were considered for these correlations (*n* = 20). Associations were calculated using Spearman rank correlation and are shown with Pearson trend lines for visualization. **d**,**e**, Opt-SNE projections of concatenated cytometry data for activated Ki67^+^CD38^+^ CD4 (**d**) and activated Ki67^+^CD38^+^ CD8 (**e**) T cells for each time point and group combination are shown. Groups: healthy controls (*n* = 10); MS-aCD20 with anti-RBD IgG^+^ at any time point (MS-aCD20 RBD antibody^+^, *n* = 10); and MS-aCD20 with anti-RBD IgG^−^ at all time points examined (MS-aCD20 RBD antibody^−^, *n* = 10). **f**, AIM^+^ frequencies of the indicated T cell populations after mRNA vaccination at T4 and T5. The values represent the background-subtracted frequency of AIM^+^ non-naive T cells above paired baseline frequencies for healthy controls (gray, *n* = 10), MS RBD antibody^+^ (orange, *n* = 10) and MS RBD antibody^−^ (purple, *n* = 10) groups. Statistics were calculated using an unpaired, two-tailed Wilcoxon test. *P < 0.05, **P < 0.01.

**Extended Data Fig. 10:**
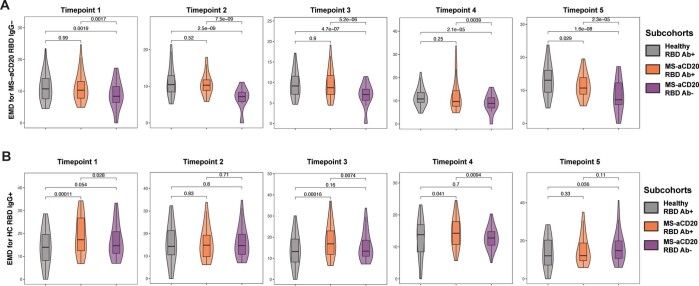
EMD analysis of activated CD4 and CD8 T cells of MS-aCD20 RBD antibody^+^ and RBD antibody^-^ patients and healthy controls after SARS-CoV-2 mRNA vaccination. **a)** Violin plots representing the summary statistics (median, distribution) of the EMD distances to MS-aCD20 RBD Ab- samples on the activated CD4 T cell opt-SNE maps across all timepoints T1-T5 for the three groups: Healthy RBD Ab+, MS-aCD20 RBD Ab+ and MS-aCD20 RBD Ab-. Pairwise comparisons of means were done with unpaired, two-tailed Wilcoxon test and *P* values are shown. **b)** Violin plots representing the summary statistics (median, distribution) of the EMD distances to Healthy RBD Ab+ samples on the activated CD8 T cell opt-SNE maps across all timepoints T1-T5 for the three groups: Healthy RBD Ab+, MS-aCD20 RBD Ab+ and MS-aCD20 RBD Ab-. Pairwise comparisons of means were done with unpaired, two-tailed Wilcoxon test and *P* values are shown. For each timepoint: Healthy RBD Ab+ (grey, n = 10), MS-aCD20 RBD Ab+ (orange, n = 10) and MS-aCD20 RBD Ab- (purple, n = 10).

We next examined how variation in the extent of B cell depletion might impact coordination with other features of vaccine-induced immune responses in patients with MS treated with aCD20. First, comparison of antigen-specific measures across T2, T4 and T5 revealed a strong correlation between humoral and circulating T_FH_ responses (Fig. 6a,b). This correlation was evident earlier and to a stronger extent in the MS-aCD20 group. By contrast, AIM^+^CD8 T cells showed a strong negative correlation with humoral immune features at T5 in the MS-aCD20 group (Fig. 6a,c). AIM^+^T_H_1 cells were also no longer positively associated with some features of humoral immunity as observed in healthy controls (Fig. 6a). These findings prompted us to separate patients with MS treated with aCD20 into those who made a detectable RBD-specific IgG response (RBD antibody^+^, *n* = 10) and those who never developed an RBD-specific IgG response (RBD antibody^−^, *n* = 10), and then investigate other potential immune differences between these two subgroups of patients. Figure 6d shows the opt-SNE projections of Ki67^+^CD38^+^ CD4 T cells for the three groups: healthy controls; MS-aCD20 RBD antibody^+^; and MS-aCD20 RBD antibody^−^. The landscape of Ki67^+^CD38^+^ CD4 T cells from RBD antibody^+^ MS-aCD20 patients was similar to that of healthy controls and both RBD antibody^+^ MS-aCD20 and healthy controls displayed some overlapping temporal features of change during the course of vaccination. By contrast, the RBD antibody^−^ MS-aCD20 group displayed a distinct opt-SNE projection of Ki67^+^CD38^+^ CD4 T cells with minimal vaccine-induced changes. To quantify these differences, we used the earth mover’s distance (EMD) metric for all pair-wise comparisons to calculate similarities between probability distributions within the opt-SNE maps. EMD revealed similarity in the overall landscape of activated CD4 T cells between healthy controls and RBD antibody^+^ MS-aCD20 patients, whereas the RBD antibody^−^ MS-aCD20 group was highly dissimilar to the other groups at all time points (Extended Data Figure 10a). By contrast to activated CD4 T cells, vaccine-induced changes in the activated CD8 T cell compartment after the first dose (T2) were more similar in RBD antibody^+^ and antibody^−^ MS-aCD20 groups, both of which resembled the healthy control responses (Fig. 6e and Extended Data Figure 10b). However, after the second vaccine dose (T4), the RBD antibody^+^ MS-aCD20 group was different from both the healthy control and RBD^−^ MS-aCD20 groups (Fig. 6e and Extended Data Figure 10b) due to the larger presence of metacluster 8. Taken together, these data show that, in the absence of a functional humoral response using anti-RBD IgG as a proxy, the defects identified in vaccine-induced responses of activated CD4 T cells in patients with MS treated with aCD20 were amplified. By contrast, vaccine-induced CD8 T cell responses were more similar in patients with MS treated with aCD20 compared to healthy controls with less impact of anti-RBD IgG status.

Finally, we assessed whether the differential vaccine responses of the CD4 and CD8 T cell subsets in patients with MS treated with aCD20 separated by RBD IgG response were related to the induction of antigen-specific T cell responses. The MS-aCD20 RBD antibody^−^ group showed markedly lower abundance of AIM^+^ circulating T_FH_ cells at T4 and T5 (Fig. 6f). By contrast, AIM^+^ T_H_1 cells were similar in RBD antibody^+^ and RBD antibody^−^ groups. Notably, AIM^+^CD8 T cell vaccine responses were significantly more robust in MS-aCD20 RBD antibody^−^ patients compared to RBD antibody^+^ patients after the second vaccine dose, supporting the notion that SARS-CoV-2 mRNA vaccine-induced CD8 T cell responses were more vigorous in patients who lacked B cells and antibody responses due to aCD20 treatment. Together, these data underscore the interrelated and coordinated nature of mRNA vaccine-induced immune responses and shed light on underlying ‘immune health’ differences in patients with MS on aCD20 therapy.

## Discussion

The goal of this study was to evaluate the impact of aCD20 therapy on SARS-CoV-2 mRNA vaccine responses. Therapy with aCD20 is used in many clinical settings including cancer immunotherapy, rheumatology and neurology. In MS, aCD20 treatment is commonly used as monotherapy offering the advantage of studying its effects in a patient population relatively less confounded by other concurrent immune therapies.

Although neutralizing antibodies are likely to be important in vaccine-induced protection, precise correlates of immunity are incompletely defined and recent evidence also points to a role for T cells^36–39,48^. Despite poor antibody responses in most patients with MS treated with aCD20, all of these patients generated robust CD4 and CD8 T cell responses to SARS-CoV-2 mRNA vaccination suggesting that vaccinating B cell-deficient patients is still likely to provide some measure of immunity to SARS-CoV-2, especially considering that T cells may retain recognition of emerging variants of concern that have escaped antibody neutralization^40,41^. Despite this preserved T cell priming, patients with MS treated with aCD20 had selective defects in antigen-specific circulating T_FH_ responses compared to healthy controls, an effect more severe in patients with MS treated with aCD20 who lacked RBD antibody responses. Although it is possible that some of these changes could reflect an impact of aCD20 on a subset of CD20^+^ T cells^49,50^, these data are also consistent with the idea that not only do T_FH_ cells provide help to B cells^51^, but that germinal center B cells also influence optimal T_FH_ cell responses^52^. By contrast, T_H_1 cell responses were only mildly impacted and CD8 T cell responses were augmented, especially after the second vaccine dose. Although these T cell responses are promising indicators of immunity in patients with MS treated with aCD20, future clinical studies examining the rate of SARS-CoV-2 infection, vulnerability to variants of concern and COVID-19 outcomes in patients with primary and secondary B cell immunodeficiencies will be necessary to fully interrogate the degree of clinical protection in these patients after mRNA vaccination.

B cell reconstitution in the circulation was, as expected, preferentially detected in patients who were farther removed from their last aCD20 treatment. This patient subgroup more efficiently generated antibodies and memory B cells against spike and RBD and had less perturbed CD4 and CD8 T cell responses to mRNA vaccination. The magnitude of vaccine-induced humoral responses correlated better with the extent of B cell reconstitution at the time of vaccination than with the time window between vaccination and the last aCD20 infusion, suggesting that the underlying mechanism for this effect is B cell reconstitution. Thus, assessing reemergence of peripheral B cells may be a better marker than time since last aCD20 treatment to determine which patients will generate humoral immunity after vaccination and future booster doses.

One unexpected finding was the more robust and functionally competent vaccine-induced CD8 T cell response after SARS-CoV-2 mRNA vaccination in the patients with MS treated with aCD20, especially patients who failed to generate anti-RBD IgG. This difference was most prominent after the administration of the second vaccine dose. These results are evidence of effective immune priming by mRNA vaccines in the absence of circulating B cells, findings that may also be relevant for the application of mRNA vaccines in other settings, such as neoantigen cancer vaccines in patients with B cell deficiencies^53^. An important question to address in the future is the underlying mechanism of this augmented CD8 T cell response. One possibility is that, in the absence of antibody, there is an increased abundance of antigen to drive CD8 T cell activation and proliferation due to lack of antigen clearance by vaccine-induced antibodies. Alternatively, regulatory B cells may play a direct role in attenuating CD8 T cell responses^54,55^. A third possibility is through the effects of antibody or immune complexes via engagement of the inhibitory Fc receptor FcγRIIB on dendritic cells^56,57^ or CD8 T cells^58^. Future studies will be necessary to determine the contribution of these possible mechanisms.

Overall, these studies provide strong evidence of immune priming by SARS-CoV-2 mRNA vaccines in patients with MS treated with aCD20. Although most of these patients do not generate optimal antibody responses, T cell priming, especially of T_H_1 and CD8 T cells, is largely intact. However, treatment with aCD20 and B cell deficiency were associated with altered coordination of the immune response and circulating T_FH_ responses were compromised. Nevertheless, despite the intent of aCD20 treatment to remove B cell-mediated immunity, including the effects of B cells in presenting antigen to CD4 T cells, these studies reveal variable levels of residual underlying immune functionality in patients with MS treated with aCD20. It will be important in the future to determine whether the residual humoral immunity and sustained or augmented T_H_1 or CD8 T cell responses, respectively, retain the ability to respond to emerging variants of concern of SARS-CoV-2. We also note that analysis of mRNA vaccine-induced immune responses serves not only to measure immunity to SARS-CoV-2 but also as an ‘analytical vaccine’ offering insights into the underlying immune health and fitness of patients with MS treated with aCD20. Overall, these data provide key insights about the ability to generate immune responses in immunocompromised populations that will be relevant for clinical guidance in these patients and possible public health recommendations for vulnerable populations.

## Methods

### Study design

In this longitudinal study, healthy controls (*n* = 10) and patients with MS treated with anti-CD20 (*n* = 20, 19 patients on ocrelizumab and 1 patient on rituximab) were recruited between December 2020 and April 2021. Plasma and PBMCs were collected immediately before the first vaccine dose (T1), 10–12 d after the first vaccine dose (T2), immediately before the second vaccine dose (T3), 10–12 d after the second vaccine dose (T4) and 25–30 d after the second vaccine dose (T5). Clinical information for healthy controls and patients with MS treated with anti-CD20 vaccinees can be found in Extended Data Fig. 1. All experiments were conducted in blinded fashion with designated members of the clinical team (who were not part of running the assays) having access to the sample key until data were collected, at which point all researchers were unblinded for the analysis. All individuals enrolled in this study provided informed written consent as part of protocols approved by the University of Pennsylvania institutional review boards and in compliance with the October 2013 Declaration of Helsinki principles. Enrolled individuals did not receive compensation for their participation in the study.

### Cell isolation and cryopreservation

Venous blood was collected in multiple 10-ml K2EDTA tubes (BD Vacutainer, catalog no. 366643; Becton, Dickinson and Company). Blood was diluted at a 1:1 ratio with PBS that contained 2 mM of EDTA and then slowly transferred to a 50-ml tube that contained 15 ml Ficoll (catalog no. CA95038-168L; GE Healthcare). Tubes were then spun at 700 *g* at room temperature with no brake. PBMC layers were collected using a transfer pipet and then washed once with 40 ml of PBS + EDTA buffer before being submitted for cell counting. Cells were then resuspended in freezer media (human AB serum + 10% DMSO) and aliquoted into cryopreserved tubes (approximately 20 million per tube). PBMC samples were first stored in Mr. Frosty freezing containers at −80 °C and then transferred to liquid nitrogen tanks for long-term storage.

### Plasma isolation

Venous blood was collected in a 10-ml K2EDTA tube. The tube was then stored upright at room temperature for 30 min before centrifugation at 4 °C for 10 minutes at 2,500 *g* (with swinging bucket rotor). Supernatants were then collected, aliquoted and stored at −80 °C until further use.

### Detection of SARS-CoV-2-specific antibodies

Plasma samples were tested for SARS-CoV-2-specific antibody by ELISA^59^. The estimated sensitivity of the test is 100% (95% confidence interval (CI), 89.1 to 100.0%) and specificity is 98.9% (95% CI, 98.0 to 99.5%). Plasmids encoding the recombinant full-length spike protein and the RBD were provided by F. Krammer and purified by nickel-nitrilotriacetic acid resin (QIAGEN). Monoclonal antibody CR3022 was included on each plate to convert optical density values into relative antibody concentrations. Plasmids needed to express CR3022 were provided by I. Wilson.

### SARS-CoV-2 neutralization assay

293T cells were transfected with pCG1 SARS-CoV-2 S D614G delta 18 expression plasmid encoding a codon-optimized SARS-CoV-2 S gene with an 18 residue truncation in the cytoplasmic tail (provided by S. Pohlmann). Twenty-four hours after transfection, the SARS-CoV-2 spike-expressing cells were infected for 2 h with vesicular stomatitis virus G pseudotyped VSVΔG-red fluorescent protein (RFP) at a multiplicity of infection of approximately 1. Media containing the VSVΔG-RFP SARS-CoV-2 pseudotypes were collected 28–30 h after infection and clarified by centrifugation. For the antibody neutralization assay using VSVΔG-RFP SARS-CoV-2, all sera were heat-inactivated for 30 min at 55 °C before use in the neutralization assay. Vero E6 cells stably expressing transmembrane protease serine 2 were seeded in a 96-well collagen-coated plate; the next day, twofold serially diluted serum samples were mixed with VSVΔG-RFP SARS-CoV-2 pseudotype virus (100–300 focus-forming units per well) and incubated for 1 h at 37 °C. Also included in this mixture to neutralize any potential carryover vesicular stomatitis virus G was 1E9F9, a mouse anti-VSV Indiana G, at a concentration of 600 ng ml^−1^ (Ab01402-2.0; Absolute Antibody). The serum-virus mixture was then used to replace the media on the Vero E6 transmembrane protease serine 2 cells. Twenty-two hours after infection, cells were washed and fixed with 4% paraformaldehyde before visualization on an S6 FluoroSpot Analyzer (CTL). Individual infected foci were enumerated and the values compared with control wells without antibody. The focus reduction neutralization titer 50% (FRNT_50_) was measured as the greatest serum dilution at which the focus count was reduced by at least 50% relative to control cells that were infected with pseudotype virus in the absence of human serum. Focus reduction neutralization titers 50% for each sample were measured in at least 2 technical replicates and were reported for each sample as the geometric mean of the technical replicates.

### Flow cytometry

Samples were acquired on a 5 laser BD FACS Symphony A5 (X50 SORP). Standardized SPHERO rainbow beads (catalog no. RFP-30-5A; Spherotech) were used to track and adjust photomultiplier tubes over time. UltraComp eBeads (catalog no. 01-2222-42; Thermo Fisher Scientific) were used for compensation. Up to 1 × 10^6^ PBMCs were acquired for each sample. All antibodies used for high-dimensional FACS analysis can be found in Supplementary Table 2. All data collection was done using the BD FACSDiva Software (version 9.0).

### Detection of SARS-CoV-2-specific memory B cells

Antigen-specific B cells were detected using biotinylated proteins in combination with different streptavidin-fluorophore conjugates^42^. Biotinylated proteins were multimerized with fluorescently labeled streptavidin for 1 h at 4 °C. Full-length spike protein (R&D Systems) was mixed with streptavidin-Brilliant Violet 421 (BioLegend) at a 10:1 mass ratio (for example, 200 ng spike with 20 ng streptavidin; approximately 4:1 molar ratio). Spike RBD (R&D Systems) was mixed with streptavidin allophycocyanin (BioLegend) at a 2:1 mass ratio (for example, 25 ng RBD with 12.5 ng streptavidin; approximately 4:1 molar ratio). Biotinylated influenza hemagglutinin pools (A/Brisbane/02/2018/H1N1, B/Colorado/06/2017; Immune Technology) were mixed with streptavidin-phycoerythrin (BioLegend) at a 6.25:1 mass ratio (for example, 100 ng hemagglutinin pool with 16 ng streptavidin; approximately 6:1 molar ratio). Streptavidin-Brilliant Violet 711 (BD Biosciences) was used as a decoy probe without biotinylated protein to gate out cells that nonspecifically bind streptavidin. Antigen probes for spike, RBD and hemagglutinin were prepared individually and mixed together after multimerization with 5 µM of free D-biotin (Avidity LLC) to minimize potential cross-reactivity between probes.

### AIM assays

PBMCs were thawed by diluting with 10 ml of warm RPMI supplemented with 10% FCS, 2 mM of L-glutamine, 100 U ml^−1^ of penicillin and 100 mg ml^−1^ streptomycin (R10) and washed once in R10. Cell counts were obtained with a Countess Automated Cell Counter (Thermo Fisher Scientific) and each sample was resuspended in fresh R10 to a density of 5 × 10^6^ cells per ml^−1^. For each condition, duplicate wells containing 1 × 10^6^ cells in 200 ml were plated in 96-well round-bottom plates and rested overnight in a humidified incubator at 37 °C and 5% CO_2_. After resting, CD40-blocking antibody (0.5 mg ml^−1^ final concentration) was added to the cultures for 15 min before stimulation and cells were subsequently stimulated for 24 h with costimulation (antihuman CD28/CD49d; BD Biosciences) and peptide megapools (CD4-S for all CD4 T cell analyses, CD8-E for all CD8 T cell analyses) at a final concentration of 1 mg ml^−1^ (refs. ^60,61^). Matched unstimulated samples for each donor at each time point were treated with costimulation alone; 20 h poststimulation, antibodies targeting CXCR3 (clone G02587, dilution 1:800, catalog no. 353716; BioLegend), CCR7 (clone G043H7, dilution 1:400, catalog no. 353234; BioLegend), CD40L (clone 24-31, dilution 1:50, catalog no. 310838; BioLegend), CXCR5 (clone RF8B2, dilution 1:100, catalog no. 565191; BD Biosciences) and CCR6 (clone G034E3, dilution 1:800, catalog no. 353432; BioLegend) were added to the culture along with monensin (GolgiStop; BD Biosciences) for a 4-h stain at 37 °C. After 4 h, duplicate wells were pooled and cells were washed in PBS supplemented with 2% FCS (FACS buffer). Cells were stained for 10 min at room temperature with Ghost Dye Violet 510 and Fc receptor blocking solution (Human TruStain FcX; BioLegend) and washed once in FACS buffer. Surface staining for 30 min at room temperature was then performed with antibodies directed against: CD4 (clone SK3, dilution 1:400, catalog no. 563550; BD Biosciences); CD8 (clone RPA-T8, dilution 1:400, catalog no. 612943; BD Biosciences); CD45RA (clone HI100, dilution 1:2,000, catalog no. 751555; BD Biosciences); CD27 (clone L128, dilution 1:400, catalog no. 612829; BD Biosciences); CD3 (clone UCHT1, dilution 1:800, catalog no. 612896; BD Biosciences); CD40L (clone 24-31, dilution 1:50, catalog no. 310838; BioLegend); CD200 (clone A18042B, dilution 1:100, catalog no. 399804; BioLegend); OX40 (clone Ber-ACT35, dilution 1:1,600, catalog no. 350012; BioLegend); CD69 (clone FN50, dilution 1:400, catalog no. 310938; BioLegend); CD107a (clone H4A3, catalog no. 328644, dilution 1:100; BioLegend); granzyme B (clone GB11, catalog no. GRB17, dilution 1:3,200; Thermo Fisher Scientific); and 41BB (clone 4B4-1, dilution 1:400, catalog no. 309810; BioLegend) in FACS buffer. Cells were washed once in FACS buffer, fixed and permeabilized for 30 min at room temperature (Foxp3/Transcription Factor Fixation/Permeabilization Concentrate and Diluent; Invitrogen) and washed once in 1× permeabilization buffer before staining for intracellular IFNγ (clone 4S.B3, dilution 1:400, catalog no. 502515; BioLegend), TNF (clone MAb11, dilution 1:800, catalog no. 12-7349-82; Thermo Fisher Scientific) and IL-2 (clone MQ1-17H12, dilution 1:500, catalog no. 500328; BioLegend) overnight at 4 °C. Cells were then washed once and resuspended in 1% paraformaldehyde in PBS before data acquisition.

All data from the AIM expression assays were background-subtracted using paired unstimulated control samples. For memory T cell and helper T cell subsets, the AIM^+^ background frequency of non-naive T cells was subtracted independently for each subset. AIM^+^ cells were identified from non-naive T cell populations. AIM^+^CD4 T cells were defined by dual expression of CD200 and CD40L. AIM^+^CD8 T cells were defined by dual expression of 41BB and intracellular IFNγ.

### High-dimensional data analysis of flow cytometry data

Opt-SNE^62^ and FlowSOM^63^ analyses were performed using OMIQ (https://app.omiq.ai/). Total CD4, activated CD4 and activated CD8 T cells were analyzed separately. Markers used for all three analyses were CD27, CD45RA, CD127, T-bet, CXCR5, CD71, CD38, CCR6, HLA-DR, CTLA-4, PD-1, CCR7, CD25, CXCR3, ICOS, CXCR4, FOXP3 and Ki67. The opt-SNE parameters were: total CD4 T cells: maximum iterations 1,000, perplexity 30, theta 0.5, seed 1234, subsampling equal between cohorts: total 3 M cells (1.5 M for healthy controls and 1.5 M for patients with MS treated with aCD20 groups); activated Ki67^+^CD38^+^ CD4 T cells: maximum iterations 1,000, perplexity 30, theta 0.5, components 2, seed 1234, subsampling equal between cohorts: total 13,822 cells (6,911 cells for healthy controls and 6,911 cells for patients with MS treated with aCD20); activated Ki67^+^CD38^+^ CD8 T cells: maximum iterations 1,000, perplexity 30, theta 0.5, components 2, seed 1234, subsampling equal between cohorts: total 54,446 cells (27,223 cells for healthy controls and 27,223 cells for patients with MS treated with aCD20).

FlowSOM was performed in all three analyses using the same markers outlined above for opt-SNE and with the following parameters: number of clusters 100; number of metaclusters 10 (activated CD4, activated CD8 T cells) or 15 (total CD4 T cells); distance metric Euclidean; and consensus metaclustering.

To group individual samples on the basis of their T cell landscape, pair-wise EMD values were calculated on the opt-SNE axes for all healthy controls and patients with MS treated with aCD20 vaccinees at all time points collected using the emdist package v.0.3-1 in R v.4.0.5^64,65^.

### Statistical analysis

Owing to the heterogeneity of clinical and flow cytometry data, nonparametric tests of association were preferentially used throughout this study unless otherwise specified. Correlation coefficients between ordered features (including discrete ordinal, continuous scale or a mixture of the two) were quantified by the Spearman rank correlation coefficient; significance was assessed by the corresponding nonparametric methods (null hypothesis: *ρ* = 0). Tests of association between mixed continuous versus nonordered categorical variables were performed by unpaired Wilcoxon test (for *n* = 2 categories). The association between categorical variables was assessed by Fisher’s exact test. All tests were performed in a two-sided manner, using a nominal significance threshold of *P* < 0.05 unless otherwise specified. Other details, if any, for each experiment are provided in the relevant figure legends. Data analysis was done with the following software: R v.4.0.5; RStudio v.1.4.1106; emdist v.0.3-1; OMIQ release 2021; and Prism v.9.1.2 (GraphPad Software). *P < 0.05, **P < 0.01, ***P < 0.001, ****P < 0.0001.

### Reporting Summary

Further information on research design is available in the Nature Research Reporting Summary linked to this article.

## Online content

Any methods, additional references, Nature Research reporting summaries, source data, extended data, supplementary information, acknowledgements, peer review information; details of author contributions and competing interests; and statements of data and code availability are available at 10.1038/s41591-021-01507-2.

## Supplementary information

Supplementary Information Supplementary Figs. 1 and 2 and Tables 1 and 2 and associated legends.

Reporting Summary

Supplementary Data 1 Raw data of all spike and RBD serologies for participants.

Supplementary Data 2 Key needed to link all participants with the publicly available FCS files (links found in the Methods). Relevant for all figures/extended data figures.

## Data Availability

Data are available in the main text, figures, extended data figures and supplementary materials. Raw FCS files can be accessed through the following links. Flow cytometry files for B cell analysis (Fig. 1, Extended Data Fig. 3): https://premium.cytobank.org/cytobank/experiments/378970; flow cytometry files for the high-dimensional analysis (Figs. 2, 4, 6 and Extended Data Figs. 4, 5, 7, 10 and Supplementary Fig. 1): https://premium.cytobank.org/cytobank/experiments/378712; flow cytometry files for the AIM T cell analysis (Figs. 3, 5, 6 and Extended Data Figs. 6 and 8): https://premium.cytobank.org/cytobank/experiments/378713. Datasets on Cytobank can be accessed via a registered account, which can be obtained by visiting the website https://www.cytobank.org. The key linking the participant IDs with the FCS filenames above is provided as a CSV file in the supplementary information. The serological information of the study participants is provided as a CSV file in the supplementary information. For any additional information on the participants, please email the corresponding author A. Bar-Or (with proper institutional review board approval, when applicable, from the requesting party) at amitbar@pennmedicine.upenn.edu.
