# Supplementary material for: Cellular and humoral immune responses following SARS-CoV-2 mRNA vaccination in patients with multiple sclerosis on anti-CD20 therapy
Source: Nat Med. 2021 Sep 14;27(11):1990–2001. doi: 10.1038/s41591-021-01507-2 (PMC8604727; doi:10.1038/s41591-021-01507-2)
Supplement: Supplementary file 1 — Supplementary Figs. 1 and 2 and Tables 1 and 2 and associated legends. [file 41591_2021_1507_MOESM1_ESM.pdf]

nature portfolio

https://doi.org/10.1038/s41591-021-01507-2

Supplementary information

Cellular and humoral immune responses following SARS-CoV-2 mRNA vaccination in patients with multiple sclerosis on anti-CD20 therapy

In the format provided by the authors and unedited

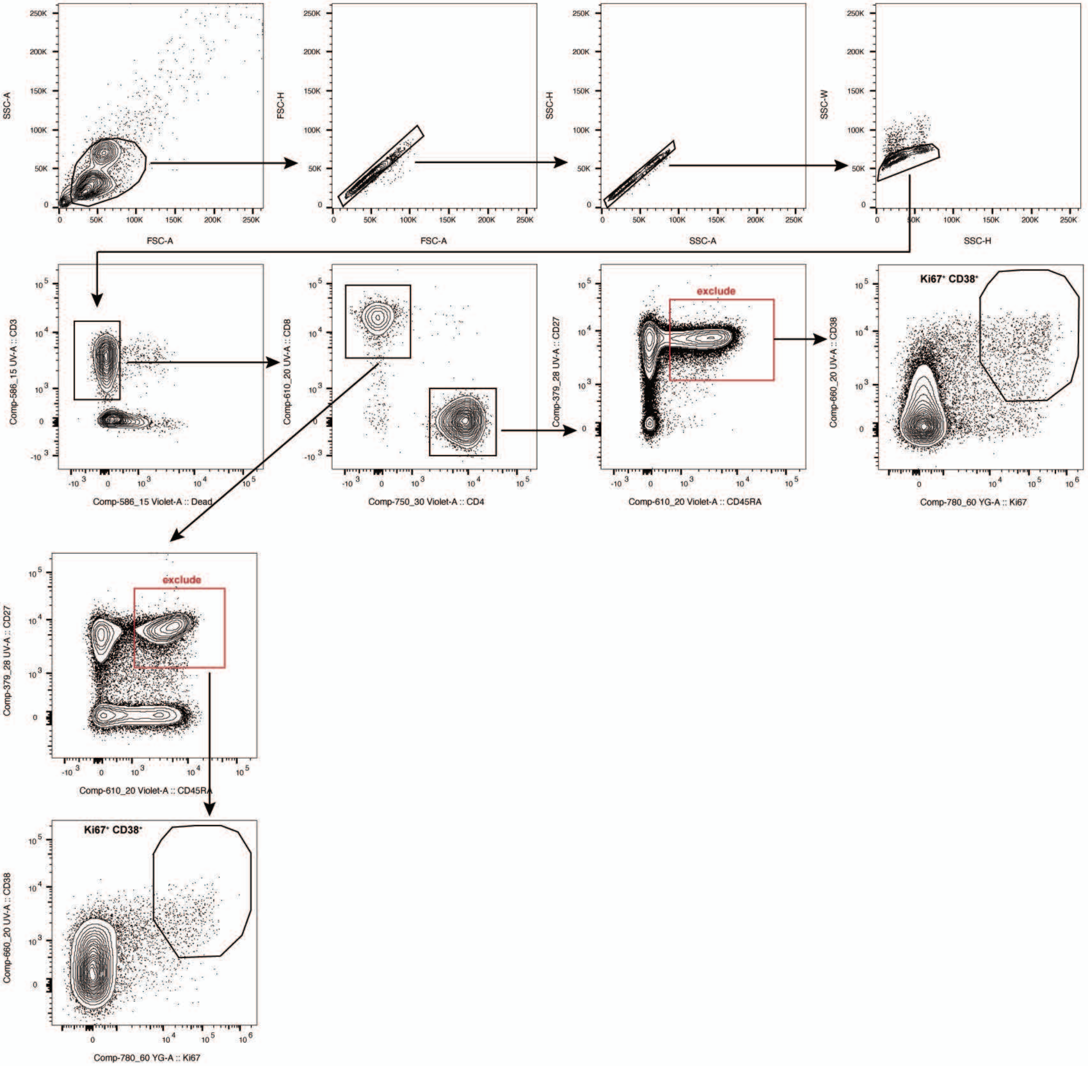
SSC-A
FSC-A
FSC-H
FSC-A
SSC-H
SSC-A
SSC-W
SSC-H
Comp-586_15 UV-A :: CD3
Comp-586_15 Violet-A :: Dead
Comp-610_20 UV-A :: CD8
Comp-750_30 Violet-A :: CD4
exclude
Comp-379_28 UV-A :: CD27
Comp-610_20 Violet-A :: CD45RA
Ki67⁺ CD38⁺
Comp-660_20 UV-A :: CD38
Comp-780_60 YG-A :: Ki67
exclude
Comp-379_28 UV-A :: CD27
Comp-610_20 Violet-A :: CD45RA
Ki67⁺ CD38⁺
Comp-660_20 UV-A :: CD38
Comp-780_60 YG-A :: Ki67

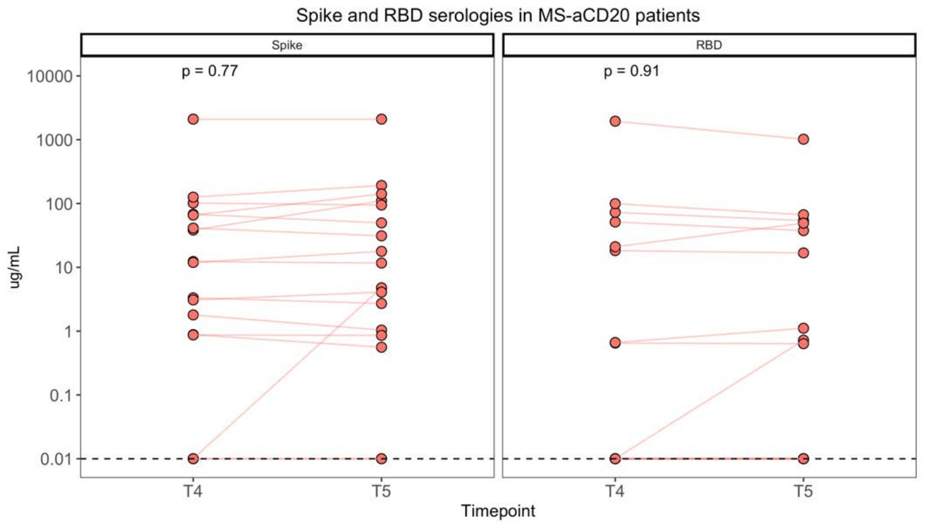
Spike and RBD serologies in MS-aCD20 patients
Spike
RBD
p = 0.77
p = 0.91
10000
1000
100
10
1
0.1
0.01
ug/mL
T4
T5
T4
T5
Timepoint

Supplementary Table 1.

Antigen-specific memory B cells	Timepoint	Comparison	p - value	method
$Spike^+$	T2	HC vs MS-aCD20	9.20E-06	Wilcoxon
$Spike^+$	T3	HC vs MS-aCD20	1.10E-05	Wilcoxon
$Spike^+$	T4	HC vs MS-aCD20	2.60E-05	Wilcoxon
$Spike^+$	T5	HC vs MS-aCD20	1.10E-05	Wilcoxon
RBD^+	T2	HC vs MS-aCD20	5.50E-06	Wilcoxon
RBD^+	T3	HC vs MS-aCD20	3.30E-06	Wilcoxon
RBD^+	T4	HC vs MS-aCD20	1.50E-05	Wilcoxon
RBD^+	T5	HC vs MS-aCD20	8.70E-06	Wilcoxon

Supplementary Table 2.

Antibody reactivity	Fluorochrome Conjugation	Clone	Supplier	Catalog #	Dilution
CD27	BUV 395	L128	BD	563815	1:200
CD71	BUV 496	M-A712	BD	750652	1:50
CD3	BUV 563	UCHT1	BD	748569	1:200
CD8	BUV 615	RPA-T8	BD	751518	1:1600
CD38	BUV 661	HIT2	BD	612969	1:200
CCR6	BUV 737	11A9	BD	612780	1:100
HLA-DR	BUV 805	G46-6	BD	748338	1:200
CTLA4	BV 421	BNI3	BD	562743	1:100
PD-1	BV 480	EH12.11	BD	566112	1:50
CCR7	BV 510	G043H7	Biolegend	353232	1:100
Zombie Yellow	BV 570	-	Biolegend	423103	1:500
CD45RA	BV 605	HI100	Biolegend	304134	1:100
CD25	BV 650	M-A251	BD	563719	1:100
CXCR3	BV 711	G025H7	Biolegend	353732	1:100
CD4	BV 750	SK3	BD	566355	1:2000
ICOS	BV 785	C398.4A	Biolegend	313534	1:50
SLAM	AF 488	A12 (7D4)	Biolegend	306312	1:50
CD127	BB 700	HIL-7R-M21	BD	566398	1:100
CXCR4	PE-Cy5	12G5	Biolegend	306508	1:500
FoxP3	PE-Cy5.5	PCH101	Fisher	35-4776-42	1:50
Ki67	PE-Cy7	B56	BD	561283	1:400
T-bet	AF 647	4B10	Biolegend	644804	1:200
CXCR5	APC-R700	RF8B2	BD	565191	1:50
Bcl-6	APC-Cy7	K112-91	BD	563581	1:100
