# Supplementary material for: Cellular and humoral immune responses following SARS-CoV-2 mRNA vaccination in patients with multiple sclerosis on anti-CD20 therapy
Source: Nat Med. 2021 Sep 14;27(11):1990–2001. doi: 10.1038/s41591-021-01507-2 (PMC8604727; doi:10.1038/s41591-021-01507-2)
Supplement: Supplementary file 2 — Reporting Summary [file 41591_2021_1507_MOESM2_ESM.pdf]

nature portfolio

Corresponding author(s): Amit Bar-Or, MD

Last updated by author(s): 8/15/2021

Reporting Summary

Nature Portfolio wishes to improve the reproducibility of the work that we publish. This form provides structure for consistency and transparency in reporting. For further information on Nature Portfolio policies, see our Editorial Policies and the Editorial Policy Checklist.

Statistics

For all statistical analyses, confirm that the following items are present in the figure legend, table legend, main text, or Methods section.

n/a	Confirmed	
☐	☒	The exact sample size (*n*) for each experimental group/condition, given as a discrete number and unit of measurement
☐	☒	A statement on whether measurements were taken from distinct samples or whether the same sample was measured repeatedly
☐	☒	The statistical test(s) used AND whether they are one- or two-sided<br>*Only common tests should be described solely by name; describe more complex techniques in the Methods section.*
☐	☒	A description of all covariates tested
☐	☒	A description of any assumptions or corrections, such as tests of normality and adjustment for multiple comparisons
☐	☒	A full description of the statistical parameters including central tendency (e.g. means) or other basic estimates (e.g. regression coefficient) AND variation (e.g. standard deviation) or associated estimates of uncertainty (e.g. confidence intervals)
☐	☒	For null hypothesis testing, the test statistic (e.g. *F*, *t*, *r*) with confidence intervals, effect sizes, degrees of freedom and *P* value noted<br>*Give P values as exact values whenever suitable.*
☒	☐	For Bayesian analysis, information on the choice of priors and Markov chain Monte Carlo settings
☒	☐	For hierarchical and complex designs, identification of the appropriate level for tests and full reporting of outcomes
☐	☒	Estimates of effect sizes (e.g. Cohen's *d*, Pearson's *r*), indicating how they were calculated

Our web collection on statistics for biologists contains articles on many of the points above.

Software and code

Policy information about availability of computer code

Data collection	All FACS-based data collection was done with BD FACS Diva Software (2019 release)
Data analysis	Data Analysis was done with the following software: R (Version 4.0.5), R Studio (Version 1.4.1106), emdist package (version 0.3-1), OMIQ (https://app.omiq.ai/, release 2021) and GraphPad Prism (Version 9.1.2)

For manuscripts utilizing custom algorithms or software that are central to the research but not yet described in published literature, software must be made available to editors and reviewers. We strongly encourage code deposition in a community repository (e.g. GitHub). See the Nature Portfolio guidelines for submitting code & software for further information.

Data

Policy information about availability of data

All manuscripts must include a data availability statement. This statement should provide the following information, where applicable:

- Accession codes, unique identifiers, or web links for publicly available datasets
- A description of any restrictions on data availability
- For clinical datasets or third party data, please ensure that the statement adheres to our policy

All data are available in the main Text, Figures, Extended Data Figures and supplementary materials. Raw .fcs files can be accessed through the following links:
- flow cytometry files for B cell analysis (Figure 1, Extended Data Figure 3):
https://premium.cytobank.org/cytobank/experiments/378970
- flow cytometry files for high-dimensional analysis (Figures 2, 4, 6 and Extended Data Figures 4, 5, 7, 10 and Supplementary Figure 1):
https://premium.cytobank.org/cytobank/experiments/378712
- flow cytometry files for AIM T cell analysis (Figures 3, 5, 6 and Extended Data Figures 6, 8)

https://premium.cytobank.org/cytobank/experiments/378713
Datasets on Cytobank can be accessed via a registered account, which can be obtained by visiting the https://www.cytobank.org/ website.
The key linking the participant IDs with the .fcs file names is provided as a .csv file in the supplementary information. The serological info of the study participants is provided as a .csv file in the supplementary information. For any additional information on the participants, please email the corresponding author Dr. Amit Bar-Or (with proper IRB approval, when applicable, from the requesting party) at amitbar@pennmedicine.upenn.edu.

Field-specific reporting

Please select the one below that is the best fit for your research. If you are not sure, read the appropriate sections before making your selection.

☒ Life sciences ☐ Behavioural & social sciences ☐ Ecological, evolutionary & environmental sciences

For a reference copy of the document with all sections, see nature.com/documents/nr-reporting-summary-flat.pdf

Life sciences study design

All studies must disclose on these points even when the disclosure is negative.

Sample size	Based on our previous studies (Goel et al, Sci Immunology, 2021) that utilized 40 healthy individuals receiving SARS-CoV-2 vaccination and based on the large effect sizes we noted at the post-vaccine timepoints (plus the published serological differences of MS patients compared to healthy volunteers following vaccination), we estimated that a total longitudinal cohort of 30 participants (20 MS and 10 healthy) would provide sufficient n to detect differences. The primary statistical goal was to compare across groups, and an F-test power calculation for three groups of 10 subjects each (which was the analysis with the fewest number of subjects per group, with three groups: HC, MS RBD IgG-, MS RBD IgG+), assuming conservatively a modest effect size of 0.6-0.7, provides a power calculation of >0.8 at an a significance level of 0.05.
Data exclusions	We excluded one patient with mutliple sclerosis who clinically had COVID-19 in the past. All healthy participants were clinically COVID-19 naive. We thus wanted to match the two groups and assess cleanly the immune response of a naive immune system to SARS-CoV-2 mRNA vaccination.
Replication	No experimental replication was conducted in this manuscript, as all samples were derived from primary human participants with a predefined longitudinal cohort study design.
Randomization	The study design did not allow for randomization (the two groups included MS patients vs HCs; all participants received mRNA vaccination). All subjects were enrolled sequentially as they agreed to participate. Covariates like age and sex were controlled by assessing these variables in both groups periodically during recruitment.
Blinding	Blinding was performed at the time of data collection. All researchers involved in data collection were blinded to the group allocation of the samples. All investigators were unblinded during the analysis to allow comparisons among the different groups.

Reporting for specific materials, systems and methods

We require information from authors about some types of materials, experimental systems and methods used in many studies. Here, indicate whether each material, system or method listed is relevant to your study. If you are not sure if a list item applies to your research, read the appropriate section before selecting a response.

Materials & experimental systems

n/a	Involved in the study	
☐	☒	Antibodies
☐	☒	Eukaryotic cell lines
☒	☐	Palaeontology and archaeology
☒	☐	Animals and other organisms
☐	☒	Human research participants
☒	☐	Clinical data
☒	☐	Dual use research of concern

Methods

n/a	Involved in the study	
☒	☐	ChIP-seq
☐	☒	Flow cytometry
☒	☐	MRI-based neuroimaging

Antibodies

Antibodies used

High-dimensional FACS:
CD27 BUV 395 Clone L128 BD Cat# 563815 Dilution 1:200
CD71 BUV 496 Clone M-A712 BD Cat# 750652 Dilution 1:50
CD3 BUV 563 Clone UCHT1 BD Cat# 748569 Dilution 1:200
CD8 BUV 615 Clone RPA-T8 BD Cat# 751518 Dilution 1:1600
CD38 BUV 661 Clone HIT2 BD Cat# 612969 Dilution 1:200
CCR6 BUV 737 Clone 11A9 BD Cat# 612780 Dilution 1:100
HLA-DR BUV 805 Clone G46-6 BD Cat# 748338 Dilution 1:200
CTLA4 BV 421 Clone BNI3 BD Cat# 562743 Dilution 1:100

PD-1 BV 480 Clone EH12.11 BD Cat# 566112 Dilution 1:50
CCR7 BV 510 Clone G043H7 Biolegend Cat# 353232 Dilution 1:100
Zombie Yellow BV 570 - Biolegend Cat# 423103 Dilution 1:500
CD45RA BV 605 Clone HI100 Biolegend Cat# 304134 Dilution 1:100
CD25 BV 650 Clone M-A251 BD Cat# 563719 Dilution 1:100
CXCR3 BV 711 Clone G025H7 Biolegend Cat# 353732 Dilution 1:100
CD4 BV 750 Clone SK3 BD Cat# 566355 Dilution 1:2000
ICOS BV 785 Clone C398.4A Biolegend Cat# 313534 Dilution 1:50
SLAM AF 488 Clone A12 (7D4) Biolegend Cat# 306312 Dilution 1:50
CD127 BB 700 Clone HIL-7R-M21 BD Cat# 566398 Dilution 1:100
CXCR4 PE-Cy5 Clone 12G5 Biolegend Cat# 306508 Dilution 1:500
FoxP3 PE-Cy5.5 Clone PCH101 Fisher Cat# 35-4776-42 Dilution 1:50
Ki67 PE-Cy7 Clone B56 BD Cat# 561283 Dilution 1:400
T-bet AF 647 Clone 4B10 Biolegend Cat# 644804 Dilution 1:200
CXCR5 APC-R700 Clone RF8B2 BD Cat# 565191 Dilution 1:50
Bcl-6 APC-Cy7 Clone K112-91 BD Cat# 563581 Dilution 1:100

AIM assays:
BUV395 CD4 BD Biosciences Clone SK3 Cat#563550 Dilution 1:400
BUV496 CD8 BD Biosciences Clone RPA-T8 Cat#612943 Dilution 1:400
BUV615 CD45RA BD Biosciences Clone HI100 Cat#751555 Dilution 1:2000
BUV737 CD27 BD Biosciences Clone L128 Cat#612829 Dilution 1:400
BUV805 CD3 BD Biosciences Clone UCHT1 Cat#612896 Dilution 1:800
BV421 CXCR3 Biolegend Clone G02587 Cat#353716 Dilution 1:800
BV650 CCR7 Biolegend Clone G043H7 Cat#353234 Dilution 1:400
BV605 CD69 Biolegend Clone FN50 Cat#310938 Dilution 1:400
BV711 CD40L Biolegend Clone 24-31 Cat#310838 Dilution 1:50
BV785 CD107a Biolegend Clone H4A3 Cat#328644 Dilution 1:100
FITC IFNγ Biolegend Clone 4S.B3 Cat#502515 Dilution 1:400
PE CD200 Biolegend Clone A18042B Cat#399804 Dilution 1:100
PE-Cy7 OX40 Biolegend Clone Ber-ACT35 Cat#350012 Dilution 1:1600
AF647 41BB Biolegend Clone 4B4-1 Cat#309810 Dilution 1:400
APC-R700 CXCR5 BD Biosciences Clone RF8B2 Cat#565191 Dilution 1:100
APC-Cy7 CCR6 Biolegend Clone G034E3 Cat#353432 Dilution 1:800
BV421 IL-2 BD Biolegend Clone MQ1-17H12 Cat# 500328 Dilution 1:500
PE-Texas Red Granzyme B ThermoFisher Clone GB11 Cat#GRB17 Dilution 1:3200
PE TNF alpha ThermoFisher Clone MAb11 Cat# 12-7349-82 Dilution 1:800

Miscellaneous:
Monoclonal antibody CR3022: plasmids to express CR3022 were provided by I. Wilson (Scripps)
Anti-VSV-G [1E9F9], clone 1E9F9, Absolute Antibody, cat# Ab01402-2.0, concentration of 600 ng/ml

Validation

All antibodies are validated by the manufacturer and are quality control tested by surface or intracellular immunofluorescent staining with flow cytometric analysis. For more information on the antibodies used, please visit biolegend.com, bdbiosciences.com and thermofisher.com.

Eukaryotic cell lines

Policy information about cell lines

Cell line source(s)

ATCC

Authentication

Quality control specifications as provided by the supplier (STR profile):
CSF1PO: 11,12
D13S317: 12,14
D16S539: 9,13
D5S818: 8,9
D7S820: 11
TH01: 7, 9.3
TPOX: 11
vWA: 16,19
Amelogenin: X

Mycoplasma contamination

All cell lines tested negative by the supplier for mycoplasma contamination

Commonly misidentified lines
(See ICLAC register)

Name any commonly misidentified cell lines used in the study and provide a rationale for their use.

Human research participants

Policy information about studies involving human research participants

Population characteristics

Among the 20 participants with multiple sclerosis, all were adults with an age range of 27-57 years (mean age of 40), with females comprising 75%. All of them had a diagnosis of relapsing-remitting multiple sclerosis, and all were being treated with

	an anti-CD20 monoclonal antibodies as monotherapy during the period of the study. All 10 participants in the healthy control group were people with no known autoimmune conditions or on any immune-modulating therapies, fell within the age range of 25-61 years (mean age of 35 years), with 60% of them being females. None of the 30 total participants included in the manuscript had a clinical history of COVID-19.
Recruitment	Participants with multiple sclerosis were recruited through the Multiple Sclerosis clinic at University of Pennsylvania Health System (UPHS). Patients who were about to get vaccinated were either identified by their primary neurologist and referred to the study recruiting team, or were identified through the vaccination clinic organized by UPHS. They were then contacted via phone or in-person and explained about the study goals, risks/benefits and requirements. Healthy controls were recruited through a word-of-mouth and group emails among employees at the University of Pennsylvania. No compensation was provided. All the participants were recruited in the early weeks to months after SARS-CoV-2 vaccinations were approved. Vaccinations at the time were being offered to participants by the 'category' they belonged to, as deemed eligible through public health and institutional recommendations. Therefore, participants were recruited in the order that they were offered vaccinations, which is unlikely to have played into selection bias by investigators. One potential means for self-selection bias could be participants' willingness for serial venipuncture, but it is unlikely that this could have impacted any of the results.
Ethics oversight	Institutional Review Board, University of Pennsylvania

Note that full information on the approval of the study protocol must also be provided in the manuscript.

Flow Cytometry

Plots

Confirm that:

☒ The axis labels state the marker and fluorochrome used (e.g. CD4-FITC).

☒ The axis scales are clearly visible. Include numbers along axes only for bottom left plot of group (a 'group' is an analysis of identical markers).

☒ All plots are contour plots with outliers or pseudocolor plots.

☒ A numerical value for number of cells or percentage (with statistics) is provided.

Methodology

Sample preparation	For all studies, PBMCs were thawed and promptly washed. Subsequently, we proceeded with either antibody staining directly or in vitro activation with peptide pools and then antibody-based staining.
Instrument	Samples were acquired on a 5 laser BD FACS Symphony A5 (X50 SORP).
Software	Events were acquired with BD FACS Diva Software (release 2019)
Cell population abundance	No sorting experiments were conducted. For FACS-based event analysis, standardized SPHERO rainbow beads (Spherotech, Cat#RFP-30-5A) were used to track and adjust PMTs over time. UltraComp eBeads (ThermoFisher, Cat#01-2222-42) were used for compensation. Up to 1x10^6 PBMCs were acquired per each sample.
Gating strategy	Gating strategies are shown in the Extended Data. Briefly, all lymphocytes were initially gated using standard FSC/SSC gating, followed up by singlet discrimination. All subsequent manual gating was done with markers optimized to have clear distinction between positive and negative populations.

☒ Tick this box to confirm that a figure exemplifying the gating strategy is provided in the Supplementary Information.
